# An in vitro paradigm to assess potential anti-Aβ antibodies for Alzheimer’s disease

**DOI:** 10.1038/s41467-018-05068-w

**Published:** 2018-07-11

**Authors:** Ming Jin, Brian O’Nuallain, Wei Hong, Justin Boyd, Valentina N. Lagomarsino, Tiernan T. O’Malley, Wen Liu, Charles R. Vanderburg, Matthew P. Frosch, Tracy Young-Pearse, Dennis J. Selkoe, Dominic M. Walsh

**Affiliations:** 1 Laboratory for Neurodegenerative Research, Ann Romney Center for Neurologic Diseases, Brigham and Women’s Hospital and Harvard Medical School, Boston, MA 02115 USA; 2 Massachusetts General Institute for Neurodegenerative Disease, Massachusetts General Hospital and Harvard Medical School, Charlestown, MA 02129 USA

## Abstract

Although the amyloid β-protein (Aβ) is believed to play an initiating role in Alzheimer’s disease (AD), the molecular characteristics of the key pathogenic Aβ forms are not well understood. As a result, it has proved difficult to identify optimal agents that target disease-relevant forms of Aβ. Here, we combined the use of Aβ-rich aqueous extracts of brain samples from AD patients as a source of human Aβ and live-cell imaging of iPSC-derived human neurons to develop a bioassay capable of quantifying the relative protective effects of multiple anti-Aβ antibodies. We report the characterization of 1C22, an aggregate-preferring murine anti-Aβ antibody, which better protects against forms of Aβ oligomers that are toxic to neurites than do the murine precursors of the clinical immunotherapeutics, bapineuzumab and solanezumab. These results suggest further examination of 1C22 is warranted, and that this bioassay maybe useful as a primary screen to identify yet more potent anti-Aβ therapeutics.

## Abstract (web summary)

The optimal Aβ species to target for the potential treatment of Alzheimer’s disease has not yet been established. Here, the authors describe an in vitro assay that uses extracts from brain tissue from patients with Alzheimer’s disease, and application to human iPSC-derived neurons, to compare the neuroprotective potential of several anti-Aβ antibodies.

## Introduction

Approaches using monoclonal antibodies to target the amyloid β-protein (Aβ) constitute the largest and most advanced therapeutic effort to treat Alzheimer’s disease (AD)^1–3^. Despite generally good outcomes in preclinical mouse models, anti-Aβ immunotherapy has yielded limited success in humans^2,3^. Explanations offered to account for the poor translation of pre-clinical lead antibodies into human therapies include imperfect trial design, intervention at a disease stage when there is already significant neural loss, and inappropriate target selectivity of the antibodies used^2,4,5^.

When assessing the efficacy of any therapeutic, there are several issues to consider besides target engagement, and yet the specific targeting of the most cytotoxic forms of Aβ is by far the most critical requirement. Synthetic Aβ can exist in vitro in a bewildering array of assemblies that differ in structure and size^6^, but it remains unclear whether the assemblies that can be formed in vitro ever exist in the human brain. In striking contrast to the hundreds of studies that have investigated the aggregation and toxicity of synthetic Aβ, only ~20 studies have focused on aqueously soluble Aβ species extracted directly from human brain. These studies can be divided into three categories: efforts to identify the primary sequence and/or assembly forms that constitute water-soluble Aβ, whether bioactive or not^7–21^; attempts to investigate the cytotoxic activity^22–27^ or seeding activity^28,29^ of crude Aβ–containing extracts; and efforts to study the assembly size of the neurotoxic components of Aβ-rich brain extracts^30–34^. Collectively, these studies suggest that Aβ in aqueous extracts of AD brain exists as a mixture of different sized assemblies^10,12–14,21,30^ and that one or more of these are extremely potent toxins^22–26,30–33^. Indeed, in some experiments, human brain-derived Aβ assemblies were found to be many orders of magnitude more potent than synthetic Aβ peptides^24,32^. Recently, we have shown that only a fraction of AD brain-derived Aβ has disease-relevant bioactivity^34^.

There are now at least 9 anti-Aβ monoclonal antibodies (mAbs) at various stages of clinical investigation^35^, five of which are believed to preferentially target Aβ oligomers^25,36–39^. Three of these advanced mAbs—crenezumab^25^, BAN2401^14,40^, and SAR228810^38^—were selected against synthetic Aβ, whereas aducanumab was selected based on immunohistochemical detection of AD amyloid plaques^37,41^. The rationale underlying the use of putatively oligomer-specific mAbs is based on the hypothesis that both Aβ monomers and insoluble fibrillar plaques are relatively innocuous; therefore, an ideal antibody would react weakly with monomers and mature fibrils, but strongly with diffusible oligomers. A key requirement for all CNS immunotherapies is delivering sufficient mAb to the brain. Normally, only ~0.1% of circulating antibody arrives in the brain at steady state^42^, so it is essential that the antibody that does enter the brain is not lost on superfluous targets. One explanation for the disappointing clinical efficacy of anti-Aβ antibodies in human trials is that they target a broad range of Aβ species, including many relatively inactive forms^34^ and thus cannot attain the necessary therapeutic concentration against bioactive forms. Similarly, in certain studies sub-optimal antibody levels were used to avoid side-effects such amyloid-related imaging abnormalities (ARIA)^43^.

Currently, there is no information in the public domain about the relative ability of candidate therapeutic antibodies to recognize toxic forms of Aβ in human brain, and the properties of optimal therapeutic Aβ antibodies remain ill-defined. To address this central problem, we generated an aggregate-preferring mAb, called 1C22, which shares many of the characteristics of the anti-oligomer mAbs in clinical development^25,36,40,44,45^, and we compared its binding properties to those of the murine precursors of solanezumab (mAb 266) and bapineuzumab (mAb 3D6). Solanezumab continues to be tested in two secondary prevention trials^46,47^, and an Fc-modified form of bapineuzumab, called AAB-003, is being assessed for treating mild AD^48^. We found that both 3D6 and 266 bound tightly to monomers, whereas 1C22 bound monomers only weakly, and that 1C22 preferentially bound protofibrils (PFs) of Aβ. PFs comprise a heterogeneous mixture of prefibrillar assemblies which by EM appear as short flexible rods with an average width of 5.8 ± 0.2 nm and length <300 nm^49,50^.

Having established that 1C22, 3D6 and 266 possess distinct binding preferences, we examined the most important property of any potential anti-Aβ immunotherapeutic, its ability to neutralize neurotoxic Aβ. For this purpose, we developed a sensitive medium-throughput assay based on the application of Aβ-containing extracts from AD brain to iPSC-derived human neurons. The Aβ-containing extracts induced a concentration- and time-dependent degeneration of neurites that was attenuated by each of the 3 anti-Aβ mAbs. 1C22 and 3D6 produced effective dose-dependent protection against bioactive human Aβ with apparent IC_50_s of ~0.8 and 1.1 ng/ml, respectively. However, the protection afforded by 266 was so modest that it was not possible to estimate an IC_50_. Thus, the paradigm described here can quantitatively differentiate mAbs based on their ability to neutralize human neurotoxic Aβ. These results recommend this paradigm as a primary screen to identify even more potent anti-Aβ therapeutics, and they suggest that further examination of 1C22 is warranted.

## Results

The study of Aβ aggregation and antibodies that bind to Aβ aggregates is complicated by the fact that multiple Aβ species exist in a dynamic equilibrium^6,51^. In order to produce soluble aggregates of Aβ free of both Aβ monomer and fibrils, we used a covalently-stabilized synthetic Aβ dimer, [Aβ1-40S26C]_2_, that readily assemblies to form kinetically trapped, soluble protofibrils (PFs) (Supplementary Figure 1)^49,50^. Aggregate-free wild-type monomers were isolated by size exclusion chromatography (Supplementary Figure 1). 1C22 was generated by immunizing mice with [Aβ1-40S26C]_2_ and using a four-step screen to identify antibodies that preferentially recognize Aβ aggregates (Supplementary Figure 2). From an initial pool of ~ 7000 hybridomas, we selected 1C22. Thereafter, we compared the ability of 1C22, 3D6 and 266 to bind to synthetic Aβ monomer and kinetically trapped Aβ PFs.

### 1C22 preferentially binds to Aβ aggregates

**Fig. 1:**
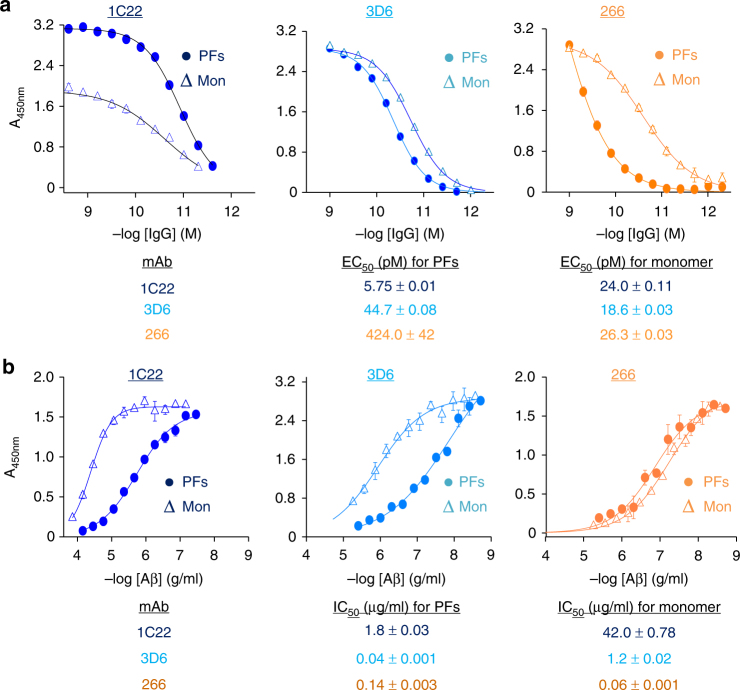
MAb 1C22 binds to PFs better than monomer. **a** Aβ1-40 monomer (Mon) and [Aβ1-40S26C]_2_ protofibrils (PFs) were immobilized at 200 ng/well on microtiter plates and mAbs 1C22, 3D6 and 266 diluted across the plates. Antibody binding curves were sigmoidally fit and used to determine the concentration of antibody that gave half-maximal binding EC_50_. Values in the table are in pM. **b** Aβ competition curves for mAbs binding to plate-immobilized Aβ monomers in the presence or absence of solution-phase Mon or PFs competitors were sigmoidally fit and used to determine the concentration of competitor that produced half-maximal inhibition of mAb binding (IC_50_). Values in the table are in μg/ml. In both **a** and **b** values are the average±SD of each condition analyzed in triplicate. When error bars are not visible they are smaller than the size of the symbol. Because we do not know the molecular weight of protofibrils the concentration of Aβ is given in μg/ml. In contrast, since the molecular weight of IgG is known, we provided mAb concentration in molar amounts. All Aβ concentrations are based on monomer molar equivalents and results are representative of at least 3 independent experiments

Initial experiments focused on the binding of mAbs to plate-immobilized synthetic Aβ. Monomers and PFs were immobilized at a constant concentration (200 ng/well), and 1C22, 3D6, and 266 were diluted across the plates. Each mAb produced a sigmoidal titer curve for both Aβ monomers and PFs (Figure 1a). 3D6 exhibited comparable binding to both Aβ monomers and PFs, with half maximal binding (EC_50_) achieved at antibody concentrations of ~40 and ~20 pM, respectively. In contrast, 266 exhibited significantly stronger binding for monomers (EC_50_~30 pM) than PFs (EC_50_ ~420 pM). 1C22 showed the reverse preference, binding more tightly to PFs (EC_50_ ~6 pM) than to monomers (EC_50_ ~20 pM). Thus, with regard to their ability to bind surface-immobilized Aβ, there was a clear difference between the 3 mAbs, with 1C22 showing tighter binding to PFs, 266 binding better to monomers, and 3D6 exhibiting only a marginal preference for PFs.

To explore the relative reactivity of mAbs with Aβ in solution, we modified our direct ELISA to measure binding to plate-immobilized monomer in the presence or absence of solution-phase Aβ conformers. By holding the concentration of mAb and plate-immobilized Aβ monomer constant and adding increasing amounts of competing soluble monomer or soluble PFs, we estimated the relative preference for the mAbs to bind solution-phase Aβ conformers. For all mAbs, addition of solution-phase Aβ caused a concentration-dependent inhibition of binding to plate-immobilized monomer (Fig. 1b). When 266 was tested, both monomer and PFs caused a similar level of inhibition, with half-maximal inhibition (IC_50_) achieved at monomer and PF concentrations of ~0.06 and ~0.14 μg/ml, respectively. In contrast, binding of both 1C22 and 3D6 to plate-immobilized Aβ monomer was more effectively competed by solution-phase PFs than solution-phase monomer. The competition ELISA (Fig. 1b) and direct ELISA (Fig. 1a) results for 1C22 indicate that this antibody has a clear preference for both immobilized and solution-phase PFs. However, for 3D6 and 266 the competition ELISA and direct ELISA yielded somewhat divergent results. 3D6 showed a modest preference for immobilized monomers, but a significant preference for solution-phase PFs. Similarly, 266 shows a strong preference for immobilized monomer, but only a slight preference for solution-phase monomer.

**Fig. 2:**
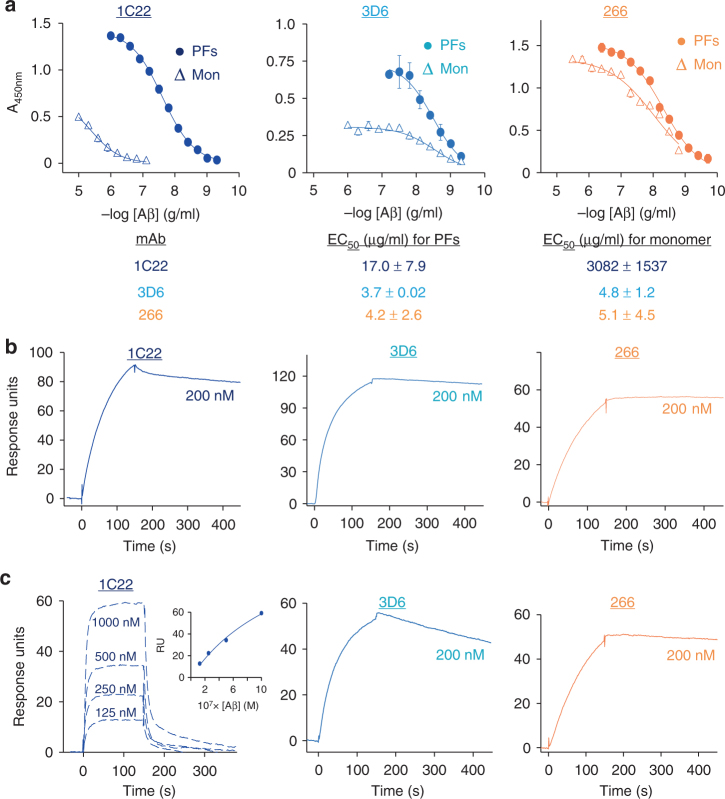
Surface-immobilized 1C22 mAb preferentially binds to PFs in solution. **a** mAbs 1C22, 3D6, and 266 were immobilized on the wells of microtiter plates and allowed to bind solution-phase protofibrils (PFs) and Aβ monomers (Mon). Antibody binding curves were sigmoidally fit and used to determine the concentration of antibody that gave half-maximal binding, EC_50_. When error bars are not visible they are smaller than the size of the symbol. Values in the table are in μg/ml and are the average ± SD of each condition analyzed in triplicate. Antibodies were immobilized on CM5 chips and solution-phase **b** PFs, or **c** Mon added. The molar concentration of Aβ monomers and PFs (wrt to Aβ monomer content) used is indicated on each sensogram. Except for Aβ monomer binding by 1C22, sensograms for mAb binding to Aβ monomers were fit to a 1:1 langmuir binding model. Sensograms for 1C22 binding to Aβ monomers were fit to steady state analysis. The inset in **c** panel 1 is a plot of response units (RU) at steady state for Aβ monomers binding to chip-immobilized 1C22. The apparent binding constant (*K*_APP_) of mAbs for PFs are: 1C22 < 1 nM; 3D6 < 1 nM; 266 < 1 nM, and the binding constants (*K*_D_) for Mon are: 1C22 = 1100 ± 500 nM; 3D6 = 7.9 ± 0.16 nM; and 266 = 2.1 ± 1.8 nM

Since immobilization of monomer could lead to surface-induced conformational changes, molecular crowding and/or aggregation, we also investigated mAb binding to monomers and PFs when the mAbs were immobilized and the Aβ conformers were in solution. In these experiments, all 3 antibodies exhibited similar strong binding to soluble PFs, but differed in how well and how much soluble monomer they bound (Fig. 2a). 266 exhibited comparable binding to soluble PFs and monomer with EC_50_s of ~4.2 and ~5.1 ng/ml, respectively (Fig. 2a, right panel). 3D6 also exhibited comparable binding to soluble PFs (EC_50_~3.7 ng/ml) and monomer (EC_50_~4.8 ng/ml) although the maximal signal was much greater for PFs than monomer (Fig. 2a, middle panel). Notably, 1C22 again showed by far the weakest interaction with monomer (EC_50_~3082 ng/ml; Fig. 2a, left panel). Based on these findings 1C22 stands out as the only mAb tested that exhibits strong preferential binding to PFs—whether immobilized or in solution. In contrast, 266 bound immobilized PFs weakly, but bound to both PFs and monomer in solution similarly well (Fig. 2a, right panel). We hypothesize that the results in Figs. 1b, 2a are more comparable than those in Fig. 1a because the former are measuring relative binding to conformers in solution, whereas surface immobilizations of Aβ conformers leads to loss of relevant epitopes through conformational changes and/or molecular crowding.

Like the direct and competition ELISAs, the capture ELISA (with mAbs immobilized) has certain limitations. For instance, epitope access by the detector anti-Aβ antiserum (AW7) may be differentially influenced by the sites at which immobilized 1C22, 266 and 3D6 bind solution-phase conformers. Thus, we also used surface plasmon resonance (SPR), a label-free, real-time technique ideal for directly measuring antibody-antigen binding. When mAbs were conjugated to SPR chips, all 3 mAbs irreversibly bound to PFs presented in solution with *K*_D_(app)’s <1 nM (Fig. 2b). Similarly, 266 and 3D6 bound soluble monomer with very high affinity and barely measureable off-rates (Fig. 2c). Since mAbs 266 and 3D6 form very tight complexes with Aβ monomers, it is only possible to determine apparent *K*_D_ values (2.1 ± 1.8 nM for 266; 7.9 ± 0.16 nM for 3D6, values are means ± SD, with *n* ≥ 3). Moreover, there is currently no appropriate model to determine the binding avidity of mAbs to PFs. Nevertheless, it is clear from inspection of the SPR sensograms for soluble PF and monomer binding that 266 and 3D6 bound immeasurably stronger to PFs because of a relatively slow rate of dissociation of the mAb–PFs complexes (Fig. 2c). In contrast, 1C22 binding of monomer had measurable on- and off-rates with a calculated *K*_D_ of 1.1 ± 0.5 μM (Fig. 2c, left graph). Overall, these SPR results are in good agreement with those obtained above using ELISA-based methods and indicate that 1C22 and 266 have very different antigen preferences. 1C22, which is reminiscent of BAN2401^45^, has only weak affinity for monomer but binds strongly to PFs whether in solution or immobilized. 266 has very high affinity for monomer, but binds PFs in solution to a similar level as 1C22 (Figs. 1, 2). 3D6 seems intermediate between 1C22 and 266, exhibiting tight binding to monomer in all assays but showing a slight preference for solution-phase PFs (Figs. 1, 2).

### Avidity drives the preferential binding of 1C22 to PFs

**Fig. 3:**
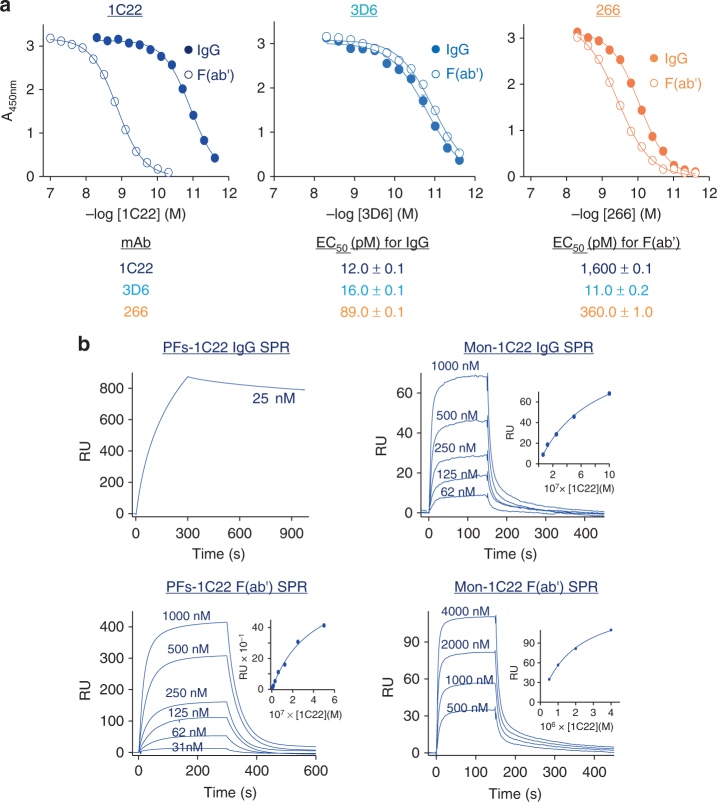
Bivalency drives 1C22 binding to PFs. **a** IgG and Fab binding curves for 1C22, 266, and 3D6 against plate-immobilized protofibrils (PFs). EC_50_ values determined from the sigmoidally fit curves demonstrated that 1C22 IgG had ~130-fold stronger reactivity against PFs than the 1C22 Fab fragment. In contrast, Fab fragments of 3D6 and 266 bound to PFs as strongly as the intact molecules. When error bars are not visible they are smaller than the size of the symbol. Values in the table are in pM and are the average ± SD of each condition analyzed in triplicate. **b** Representative sensograms for 1C22 IgG (upper panels) and Fab (lower panels) binding to CM5 chip-immobilized PFs (right panels) and monomer (Mon) (left panels) confirm that intact 1C22 binds more tightly to immobilized PFs than 1C22 Fab, whereas intact 1C22 and Fab bind similarly to Aβ monomer. Insets show plots of RU values at steady state for intact 1C22 and 1C22 Fab binding to PFs or Mon. The apparent binding constant of 1C22 IgG for PFs = 0.48 ± 0.002 nM, whereas the binding constant (*K*_D_) for 1C22 IgG with Mon = 1.39 ± 0.46 μM, and the *K*_D_ for IgG Fab binding to PFs and Mon are: 0.80 ± 0.88 μM,1.14 ± 0.49 μM, respectively

A possible explanation for why an antibody may weakly bind Aβ monomer but tightly bind Aβ aggregates (e.g. PFs) derives from the fact that IgGs have 2 identical antigen binding sites, and Aβ aggregates contain multiple identical subunits (monomers). Thus, even though the affinity of a given antigen binding site is the same for both a monomer and aggregate, because an aggregate contains multiple binding sites in close proximity, there is a high probability that the bivalent IgGs will be bound at two antigen sites. In this case, when an antibody dissociates from one site on an aggregate, it can more rapidly bind to a nearby site in a manner not possible with individual Aβ monomers. A common way to test for such enhanced binding due to avidity is to compare the binding of a monovalent form of the antibody to the intact bivalent IgG^52^. Here, we compared the binding to plate-immobilized PFs by the intact mAb vs. the Fab of the same mAb. The binding of intact mAbs to PFs was highly similar to that seen in our previous direct ELISA experiments (compare Fig. 3a vs. Fig. 1a). When the Fab of 3D6 or 266 was tested for binding to plate-immobilized PFs, the results were similar to those obtained for intact IgGs (Fig. 3a, middle and right panels). In striking contrast, the Fab of 1C22 showed dramatically reduced binding to PFs compared to the intact 1C22 IgG (Fig. 3a, left panel and Table).

For reasons detailed above, SPR has certain advantages over indirect ELISA-based modalities, so we used SPR to further compare binding of intact 1C22 and 1C22 Fab to chip-immobilized Aβ monomers or PFs. Actual and apparent *K*_D_ values determined from the fitted sensograms showed that intact 1C22 IgG bound to PFs ~1000-fold stronger (*K*_app_ = 0.48 ± 0.002 nM) than its Fab (*K*_D_ = 800 ± 88 nM) (Fig. 3b, left panels). In contrast, the intact antibody and Fab fragment showed highly similar binding to chip-immobilized monomer, with *K*_D_ values of 1.39 ± 0.46 μM and 1.14 ± 0.49 μM, respectively (Fig. 3b, right panels). These results indicate that the tight binding 1C22 displays for PFs is largely driven by avidity effects. However, this appears not to be due to repetitive display of a simple short linear epitope. Specifically, when tested for binding to a nested set of short overlapping Aβ peptide fragments, 1C22 showed only marginal binding to the fragments and much greater binding to the intact monomer (Supplementary Figure 3A, left panel, and Supplementary Figure 3B). In contrast, the well characterized N-terminal directed mAb, 6E10^53^, showed excellent binding to N-terminal fragments that contained its known epitope (Supplementary Figure 3A, right panel, and Supplementary Figure 3B). These results suggest that the relative preference of 1C22 for soluble aggregates is in part due to a requirement for an extended or conformational epitope.

### A new paradigm to assess the potential of anti-Aβ antibodies

The studies described above demonstrate that 1C22, 3D6, and 266 differ significantly with regard to their ability to bind synthetic Aβ conformers. However, given that the nature of cytotoxic Aβ in the AD brain is poorly understood, binding studies using synthetic Aβ may not accurately predict the optimal properties of antibodies intended for use in humans. Moreover, the artificial surface-immobilization of antibodies or Aβ species may give rise to avidity effects not replicated in brain when both Aβ and antibody are in solution. Thus, we sought to develop a bioactivity assay using the most disease relevant form of Aβ, namely Aβ extracted from AD brain tissue, and apply this material to human neurons in the presence and absence of antibodies.

Neuritic dystrophy is a well-accepted feature of AD^54,55^, and previously we showed that Aβ extracted from human AD cerebral cortex can disrupt the microtubule cytoskeleton of primary rat hippocampal neurons and cause time-dependent neuritic degeneration and tau phosphorylation^32^. However, the methodology we used was laborious and not suitable for testing large numbers of samples and conditions. Moreover, since we observed that rat neurons transduced to express human tau were more susceptible to the effects of the AD brain-derived Aβ (32), and it has recently been reported that human neurons are uniquely sensitive to Aβ^56^, we thought it was important to use human rather than rodent neurons. These considerations encouraged us to develop a medium throughput assay to routinely measure the neuritotoxicity of AD brain extracts on human neurons, i.e., an all human-derived bioassay.

**Fig. 4:**
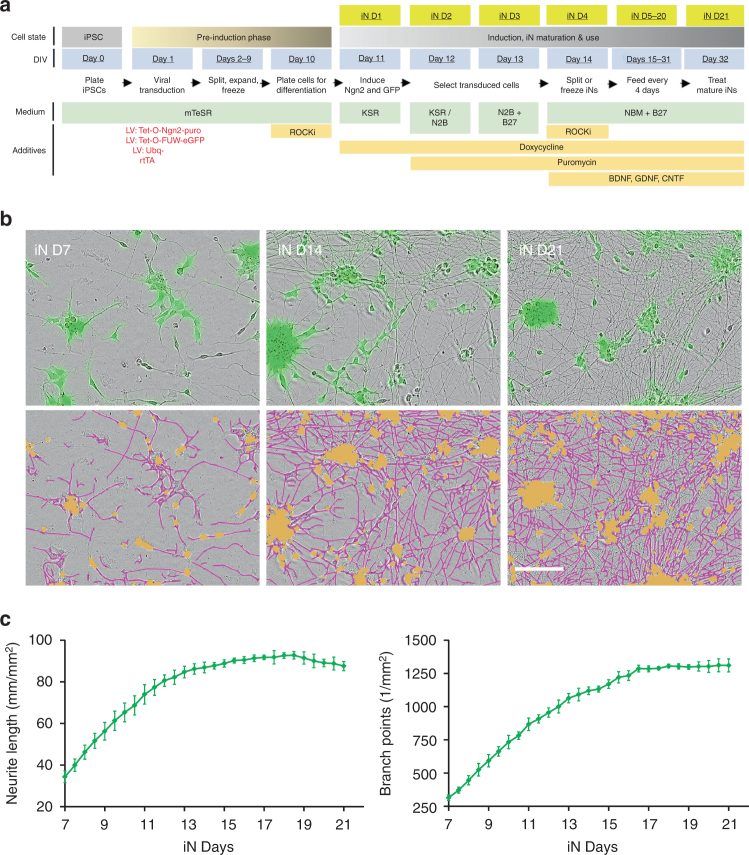
Time-lapse imaging of differentiated human induced neuron (iNs). Human induced neuron (iNs) were prepared as described in the Supplementary Methods and used for live-cell imaging from iN day 0 to iN day 21. **a** Schematic depicts the process used to generate and mature iNs and indicates the nomenclature used to designate the different stages of the process. **b** Phase contrast and fluorescence images of iN days 7, 14, and 21 are shown in the upper panels. These images were then analyzed using the IncuCutye NeuroTrack algorithm to identify neurites (pink) and cell bodies (brown). NeuroTrack-identified neurites (pink) and cell bodies (brown) are shown superimposed on the phase contrast image in the lower panels. The Scale bar is 100 μm. **c** Images were collected at 12 h intervals from iN days 7–21 and analyzed using the IncuCutye NeuroTrack algorithm to determine neurite length (left) and the number of neurite branch points (right). Each data point is the average of measurement from 12 wells of iN cells cultured in the same 96 well plate. Error bars are SEM

We took advantage of recent advances in iPSC biology to generate highly differentiated human neurons (Fig. 4) that can be prepared just as rapidly as mature rodent primary neurons. The method employed is a modified version of the Neurogenin 2 (Ngn2) differentiation protocol pioneered by the Sudhof group^57^ and is described in Supplementary Methods and illustrated in Fig. 4a. The Ngn2 method incorporates a GFP expression cassette, so all successfully transduced cells are GFP fluorescent (Fig. 4b). To assess neuritic maturation and the effects of AD brain derived Aβ on neurites, we used the IncuCyte Zoom live-cell video microscopy system from Essen Bioscience (Fig. 4b, c). Beginning 7 days after induction of Ngn2 expression (a time point we designate as iN day 7), cells were imaged every 12 h for a total of 14 days, and neurite length and branch points determined. From iN day 7–14, neurite length and branch points increased rapidly but thereafter remained constant (Fig. 4b, c). The levels of GluA1, PSD-95, synaptophysin, synapsin 1, and tau increased between iN days 7 and 14 and then remained constant (Supplementary Figure 5B). Neurons stained at iN day 21 were positive for the neuronal markers MAP2, NeuN and tau (Supplementary Figure 5C).

**Fig. 5:**
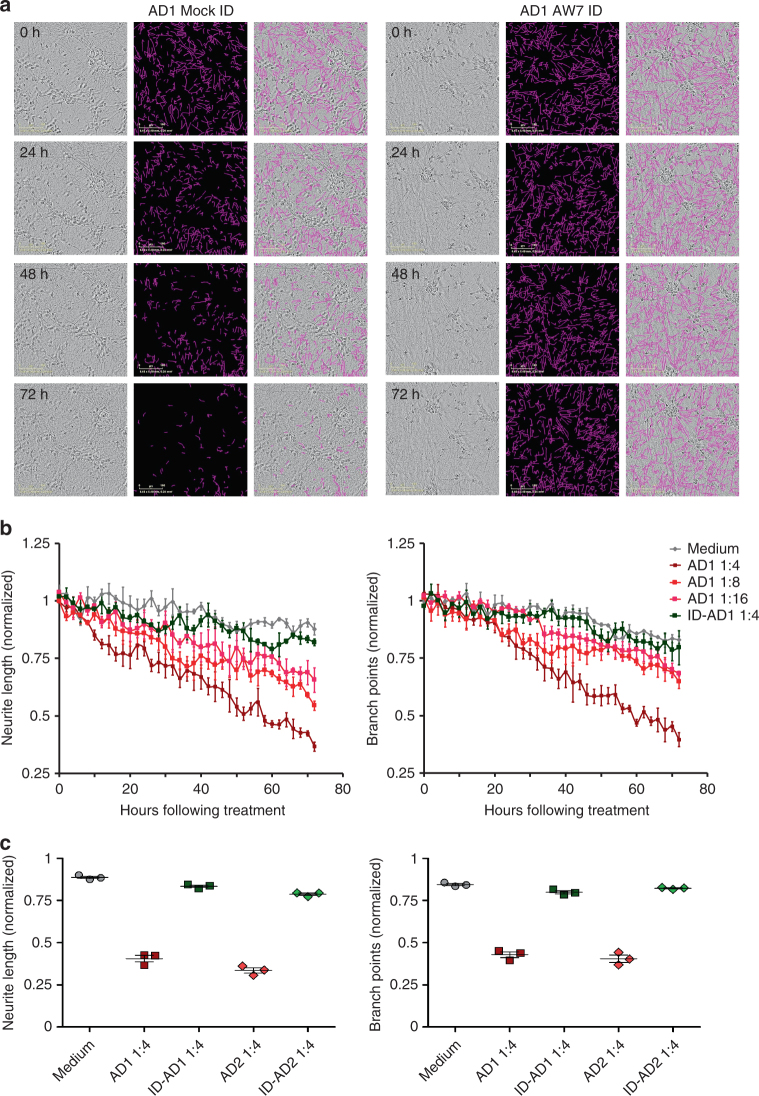
Treatment of iNs with AD brain-derived soluble Aβ induces neuritic dystrophy. Live-cell imaging was used to monitor the effect of Aβ-containing AD brain extracts on iNs. **a** iN day 21 cultures were treated with mock-immunodepleted AD1 extract (Mock ID) or AD1 extract immunodepleted with the pan anti-Aβ antiserum AW7 (AW7 ID) and cells imaged for 72 h. Phase contrast images (left panels) at 0, 24, 48, and 72 h were analyzed using the IncuCutye NeuroTrack algorithm to identify neurites (middle panels) and the NeuroTrack-identified neurites (pink) are shown superimposed on the phase contrast image (right panels). Scale bars are 100 μm. **b** Each well of iNs was imaged for 6 h prior to addition of sample and NeuroTrack-identified neurite length and branch points used to normalize neurite length and branch points measured at each interval after addition of sample. Mock-ID AD1 extract was tested at 3 dilutions, 1:4, 1:8, and 1:16. Immunodepleted AD1 was tested at 1:4 and cells treated with medium alone were used to monitor the integrity of untreated cells. The values shown in graphs are the average of triplicate wells for each treatment ± SEM. **c** Plots of normalized neurite length (left panel) and neurite branch points (right panel) are derived from the last 6 h of the traces shown in **b** and in Supplementary Figure 8B and are presented as mean values ± SEM. Application of AD1 brain extract caused a decrease in both neurite length and branch points relative to: (i) the same neurons prior to treatment, and (ii) sister wells of untreated neurons (neurite length, *p* < 0.0001; branch points, *p* < 0.0001, two-way ANOVA). Importantly, AD1 extract that had been immunodepleted of Aβ had no significant effect on either neurite length or branch points (neurite length, *p* = 0.7195; branch points, *p* = 1.0000, two-way ANOVA). The results shown are representative of at least three independent experiments

Once these consistent results were established, we exposed the neurons to Aβ-rich soluble AD brain extracts at iN day 21 and imaged every 2 h for a total of 72 h of exposure. Application of AD1 brain extract (Supplementary Figure 6A-C) caused a time- and dose-dependent decrease in both neurite length and branch points relative to the same neurons measured between −6 and 0 h prior to treatment, and sister wells of untreated neurons (neurite length, *p* < 0.0001; branch points, *p* < 0.0001, two-way ANOVA) (Fig. 5a, b). Importantly, AD1 extract that had been immunodepleted of Aβ (called ID-AD1; Supplementary Figure 6A-C) had no significant effect on either neurite length or branch points (Fig. 5b, c) (neurite length, *p* = 0.7195; branch points, *p* = 1.0000, two-way ANOVA). The effects of AD extracts were clearly dose- and Aβ-dependent irrespective of whether normalized means of triplicate wells (Fig. 5) or individual wells (Supplementary Figure 7A), or non-normalized means were used (Supplementary Figure 7B). To examine the generalizability of this effect, we tested a soluble Aβ-rich extract from a second AD brain, AD2 (Supplementary Figure 6D-F). As with AD1, AD2 caused a time- and dose-dependent decrease in both neurite length and branch points (neurite length, *p* < 0.0001; branch points, *p* < 0.0001, two-way ANOVA), whereas ID-AD2 had no effect (Supplementary Figure 8A, B and Fig. 5c) (neurite length, *p* = 1.0000; branch points, *p* = 0.9973, two-way ANOVA). Importantly, neither AD1 nor AD2 evinced any sign of overt perikaryal loss, and the number of cell body clusters remained constant throughout the course of the experiments and did not differ from the corresponding ID-AD or media controls (Supplementary Figure 8C) (AD1, AD vs ID, *p* = 1.0000; AD2, AD vs ID, *p* = 0.0745, two-way ANOVA). In separate studies, Aβ-rich brain extracts from three other AD patients also caused neuritotoxicity, albeit to different extents, and in each case neuritoxicity was prevented by specific immunodepletion of Aβ-[34]. Importantly, extracts from 2 control brains (Supplementary Figure 9) had no measurable adverse effects on iNs.

### 1C22 protects against Aβ toxicity better than 3D6 or 266

**Fig. 6:**
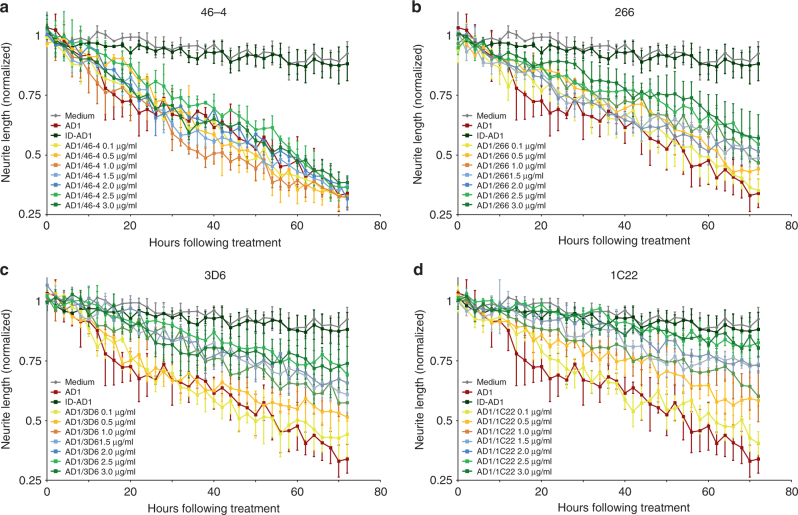
Anti-Aβ antibodies dose-dependently attenuate the neuritotoxic effects of AD brain extracts. To determine whether anti-Aβ antibodies could protect against the neuritotoxicity induced by Aβ-containing AD brain extracts iNs were treated with AD1 extract at a dilution of 1:4 in the presence or absence of increasing amounts of antibody. Graphs show time-course measurements of NeuroTrack-defined neurite length of iNs treated ± AD1 extract and **a** 46-4, **b** 266, **c** 3D6, and **d** 1C22. Each data point is the average of 3 wells±SEM

Having established a quantitative paradigm to monitor neuritotoxicity, we next assessed whether the 3 mAbs we had characterized for Aβ binding could attenuate neuritotoxicity induced by AD brain-derived Aβ. To control for non-specific antibody effects, we used an isotype control IgG1 antibody, 46–4, which was raised to HIV glycoprotein 120^58^. Half of the medium on iNs was removed (leaving ~100 μl) and then 50 μl of mAb stock solution (0.4 to 12 μg/ml) was added plus either 50 μl AD extract or fresh medium. The mAb concentrations tested were 0.1, 0.5, 1.0, 1.5, 2.0, 2.5, and 3 μg/ml and were applied in the presence or absence of a 1:4 diluted AD brain extract (which was itself a 20% (w/v) brain extract). As before, ID-AD1 had no effect on either neurite length (Fig. 6) or branch points (Supplementary Figure 10) compared to medium alone (*p* = 1.0000, two-way ANOVA). In contrast, the 1:4 diluted AD1 extract caused a profound reduction over 72 h in both neurite length (Fig. 6) and branch points (Supplementary Figure 10) compared to the 6 h pre-treatment interval and to the medium control (*p* < 0.0001, two-way ANOVA). Co-administering 46–4 did not attenuate the neuritotoxicity induced by AD1 (Fig. 6a and Supplementary Figure10A) (*p* < 0.0001, AD1/46–4 3 μg/ml vs. medium control, two-way ANOVA), whereas, addition of 1C22 caused a dose-dependent rescue of neurite length (Fig. 6d) and complexity (Supplementary Figure 10D). Notably, at 3 μg/ml 1C22 conferred near complete protection against the effects of AD1 (Fig. 6d and Supplementary Figure 10D) (*p* = 0.9840, AD1/1C22 3 μg/ml vs. medium control, two-way ANOVA). Both 266 (Fig. 6b and Ssupplementary Figure10B) and 3D6 (Fig. 6c and Supplementary Figure10C) partially protected against the disruptive effects of AD1 extract. 3D6 was more effective than 266, and 1C22 yielded the best protection. mAbs exerted similar protective effects when co-administered along with AD2 extract, i.e., 1C22 afforded the greatest protection and 266 the least (Supplementary Figure 11).

Importantly, immunocytochemical analysis of end stage cultures used in Fig. 6 confirmed the neuritic loss seen by live cell imaging and revealed an increase in phospho-tau in neurons treated with AD1 extract vs. vehicle or ID-AD1-treated cells (Supplementary Figure 12). Addition of 1C22 at 3 μg/ml completely rescued the increase in phospho tau, whereas the same concentration of 266 only modestly attenuated tau phophorylation.

**Fig. 7:**
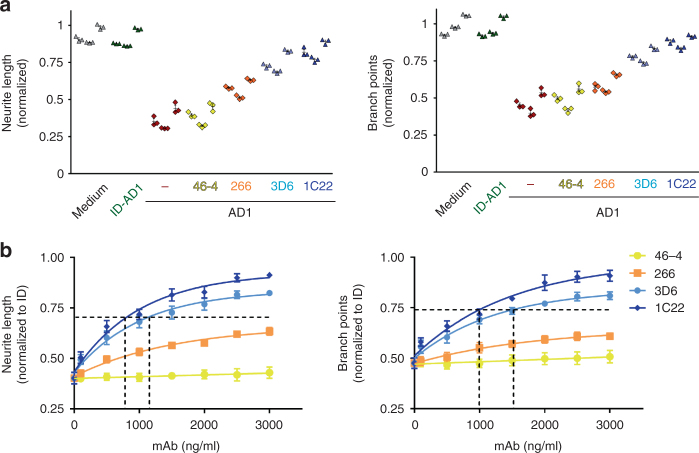
MAb 1C22 more effectively protects against AD brain extract induced neuritotoxicity than either 3D6 or 266. Three independent experiments were conducted as in Fig. 6. **a** Graphs show the normalized change in NeuroTrack-defined neurite length or branch points over the last 6 h of imaging of iNs treated with AD1 extract ± 3 μg/ml mAbs 46–4, 266, 3D6, or 1C22. In each individual experiment, 1C22 almost completely protected against the neuritotoxicity of AD1 extract and always exerted a stronger effect than 3D6, and 3D6 always provided better protection than 266 (left panel, *p* = 0.9991, 1C22 vs 3D6; *p* < 0.0001, 1C22 vs 266, two-way ANOVA). Highly similar results were obtained for neurite branch points (right panel, *p* = 0.9464, 1C22 vs 3D6; *p* < 0.0001, 1C22 vs 266, two-way ANOVA). **b** To investigate the effect of mAb concentration, NeuroTrack-defined neurite length (left panel) was averaged over the last 6 h of imaging for each treatment and values normalized to the immunodepleted AD1 treatment and neurite length plotted vs. antibody concentration. The ability of mAbs to protect neurite branch points (right panel) was determined in a similar fashion as described for neurite length. Values are the average ± SD of each condition analyzed in triplicate. When error bars are not visible they are smaller than the size of the symbol

To test the reproducibility of AD extract on neurite length and complexity, we conducted 2 replicate experiments, each time using a different iN culture and different aliquots of the AD1 extract and the mAbs, and with the experimenter always blind to the identity of the mAb. Figure 7 shows the results from the final 6 h of automated imaging for 3 separate experiments testing the effects of AD1 extract on neurite length and branch points in the presence or absence of 3 μg/ml mAb. In neurons treated with ID-AD1 or medium alone, there was a slight (but statistically insignificant) decrease in neurite length (1.1 to 13.8%), with branch point numbers sometimes slightly increased or decreased (−7.9 to 5.7 %, compared to first 6 h interval prior to treatment).

Addition of AD1 (dark red diamonds) caused a 54.6% decrease of neurite length in experiment 1, a 60.2% decrease in experiment 2, and a 46.4 % decrease in experiment 3 (Fig. 7a). Co-administration of 46–4 (yellow diamonds) did not alter neurite length relative to AD1 treatment alone (*p* = 1.0000, two-way ANOVA). In the 3 experiments, 266 (orange diamonds), 3D6 (light blue diamonds) and 1C22 (gray diamonds) protected against AD1. In each individual experiment, 1C22 always exerted a stronger effect than 3D6, and 3D6 always provided better protection than 266 (Fig. 7a left panel, *p* = 0.9991, 1C22 vs 3D6; *p* < 0.0001, 1C22 vs 266, two-way ANOVA). Highly similar results were obtained for neurite branch points (Fig. 7a right panel, *p* = 0.9464, 1C22 vs 3D6; *p* < 0.0001, 1C22 vs 266, two-way ANOVA).

To compare the dose-dependent effects of the mAbs, neurite length (Fig. 7b, left panel) was averaged over the final 6 h of imaging for each treatment and the resultant values normalized to the ID-AD1 control treatment. The results from 3 separate experiments were then averaged and plotted vs. mAb concentration and used to determine IC_50_s. 1C22 and 3D6 produced effective dose-dependent protection against AD1, with apparent IC_50_s of 0.795 μg/ml for neurite length, 0.99 μg/ml for branch points, and 1.12 μg/ml for neurite length, 1.52 μg/ml for branch points. The protection afforded by 266 was so modest that it was not possible to estimate an IC_50_.

## Discussion

Anti-Aβ immunotherapeutics are the furthest advanced among disease-modifying agents being tested in AD patients, with multiple trials underway worldwide at this writing. Until now, preclinical assessment of candidate antibodies has relied largely on in vitro binding experiments with synthetic Aβ^25,38,59^ and passive immunization of APP transgenic mice^60–62^. However, these approaches have not translated well to humans^2–4^, and it is uncertain whether synthetic Aβ peptides or APP transgenic mice can yield the type of neurotoxic Aβ assemblies that accumulate in the brains of humans with AD. In regard to behavioral deficits observed in some APP transgenic mice, it is unclear whether these are due to Aβ and/or a result of over-expression of APP^63^. Indeed, mice which produce and deposit human Aβ in the absence of over-expression of APP (i.e., APP knock-in mice or BRI2-Aβ mice) show no deficits in synaptic plasticity or memory^64^. Here we report the development of an unbiased, in vitro assay that combines the use of Aβ-rich human (AD) brain extracts and human neurons. The use of only human material in our new testing paradigm ensures that the Aβ species applied and the bioactivity readout are directly relevant to the human disease. Moreover, quantitative competition for binding to active vs. inactive forms of human Aβ is built into our system, since a large portion the Aβ species in aqueous extracts of AD brain are inactive^33,34^. While no single assay can be expected to predict the absolute utility of an anti-Aβ antibody when administered to humans, the novel paradigm described here would enable important objective comparisons of new anti-Aβ antibodies and current lead antibodies in human trials.

In addition to being based solely on human brain tissue and human neurons, our approach offers a number of other advantages over current in vivo therapeutic antibody screens. First and most obvious, our procedure is relatively rapid and should allow for the testing of large numbers of antibodies, and it could thus serve as a primary screen to identify novel antibodies of interest. Second, the measurement of neuritotoxicity and its attenuation is quantitative, making it possible to estimate the amount of antibody that would be required to neutralize neuritotoxic Aβ in the brains of patients with AD. For instance, the amount of Aβx-42 in AD1 cortical extract diluted 1:4 was ~1.55 nM and the approximate IC_50_ for mAb 1C22 was ~5.33 nM (0.8 μg/ml). Therefore, on a molar basis, a ~4-fold excess of 1C22 was required to achieve a 50% attenuation of neuritotoxicity from human Aβ. These calculations are based on measurement of Aβx-42, and analysis of a single brain extract (AD1), but it is imminently feasible to assay additional brain extracts and to measure multiple Aβ alloforms. Thus, one can readily estimate the minimal dose of any mAb required to achieve maximal binding to neurotoxic Aβ species present across a range of AD brains. Other advantages of our neuritotoxicity assay include the use of genetically identical and consistent human cultures, the supply of which is essentially limitless and could be adapted to use cells from various donors susceptible to AD. Of course, for assessing certain effects of mAbs such as ARIA^65^, in vivo animal experiments will be necessary, but this need only be done with the most promising leads identified using our screen. Such an approach would both markedly expedite the discovery process and minimize the unnecessary use of laboratory animals.

Of the three antibodies tested, our novel aggregate-preferring mAb, 1C22, was the most efficacious. Given the results of our binding studies (summarized in Supplementary Table 1) on synthetic Aβ and the prevailing belief that soluble Aβ aggregates (aka oligomers) may be the principal initiators of the AD pathogenic process^5,66–68^, the effectiveness of 1C22 vs. 3D6 and 266 may seem predictable. However, it is important to emphasize that not all soluble Aβ oligomers are bioactive^33,34^ and it is not clear if there is a specific form of Aβ that is toxic or if toxicity is conferred by a pool of soluble aggregated Aβ species. Different aggregate-preferring mAbs could exhibit distinct recognition of active vs. inactive oligomers and therefore may allow different degrees of protection against cytotoxic Aβ. Typical of the pattern we have seen in extracts from many AD brains^21,33,34^, most of the Aβ in the AD1 and AD2 water-soluble extracts was aggregated and only a small fraction existed as unaggregated monomers (Supplementary Figure 6). In vitro binding experiments indicate that 3D6 and 266 bind solution-phase synthetic PFs as well or better than 1C22 (Figs. 1–3), yet 1C22 offers the best protection against AD brain-derived neuritotoxic Aβ (Figs. 6, 7). With regard to binding to synthetic Aβ, the biggest difference between 1C22 vs. 3D6 and 266 relates to monomer. 1C22 evinces much weaker binding to monomer than either 3D6 or 266. Since, monomer contributed <7% of the total Aβ42 in the AD1 extract (Supplementary Figure 6), it seems unlikely that the differential recognition of monomer could account for the vastly superior performance of 1C22 relative to 266 in neutralizing human Aβ oligomers in our IncuCyte assay. Although, the form or forms of Aβ which mediate neuritotoxicity are as yet undefined, it is reasonable to assume that the greater protection afforded by 1C22 relative to 3D6 and 266 results from differential binding to toxic Aβ.

A concern about an avid, oligomer-preferring antibody such as 1C22 is the possibility that it will bind tightly to amyloid plaques, and that in AD brains with abundant plaques, the concentration of 1C22 available to target soluble, neurotoxic Aβ would accordingly be reduced. An ideal anti-Aβ mAb would therefore exhibit minimal reactivity with plaques^69^. Interestingly, in studies using fresh-frozen human brain (Supplementary Figure 4) and in in vivo mouse studies^70^, we have shown that 1C22 exhibits only modest binding to plaques. The relatively low binding of this highly avid antibody to plaques suggests that there is more to 1C22′s binding than avidity alone. This conclusion is consistent with our observation that 1C22 prefers an extended or conformational epitope (Supplementary Figure 3) and suggests that this epitope is present and accessible on diffusible neuritotoxic Aβ but is not readily accessible on fibrillar plaques. These results also suggest that soluble neuritotoxic Aβ oligomers may have distinct structural properties from much of the oligomeric Aβ that ends up deposited in plaques.

Currently the structural differences between naturally-occurring active and inactive Aβ oligomers are not understood, and it is only recently that the field has begun to appreciate that not all Aβ oligomers in the AD brain are equally toxic. While it will be challenging to identify the molecular bases of these differences, our new screen may aid this process. The approach described here should allow identification of monoclonal antibodies that best target brain-derived neuritotoxic Aβ, and in the future, we and others will screen libraries of small molecules to identify compounds with similarly distinct properties. In turn, the identification of such small molecules and antibodies may enable the full purification and detailed biochemical analysis of the most noxious forms of human Aβ. Focusing on relevant bioactivity assays and sources of natural (AD) Aβ, as done here, should enable the discovery of more selective and efficacious anti-Aβ immunotherapeutics as well as imaging agents and other diagnostic tools.

## Methods

### Peptides and reagents

Human Aβ(1–40) and Aβ(1–42), and Aβ(1–40) in which serine 26 was substituted with cysteine were synthesized and purified by Dr. James I. Elliott at Yale University (New Haven, CT). Peptide masses and purities ( > 95%) were confirmed by electrospray ionization/ion trap mass spectrometry and reverse-phase HPLC. Overlapping Aβ peptide fragments were synthesized and purified at the Bioploymer Laboratory in the Department of Neurology at UCLA. All other chemicals were of the highest purity available and unless indicated otherwise were obtained from Sigma-Aldrich (St. Louis, MO).

### Antibodies

The antibodies used in this study and their sources are described in Supplementary Table 2.

### Assays used to assess antibody binding to Aβ conformers

The preparation of Aβ conformers, generation and characterization of 1C22 are described in Supplementary Methods. Three distinct immunoassay formats were employed to investigate binding of mAbs to different Aβ conformers. Each assay used the same microtiter plates (#3369, COSTAR, Corning, NY), blocking buffer and assay buffer. The blocking buffer was 1% (w/v) BSA in PBS, pH 7.4, and assay buffer contained blocking buffer supplemented with 0.05% (v/v) tween 20.

Direct ELISA was performed using microtiter plate wells coated with 200 ng of Aβ conformer and subsequently blocked. Antibody binding curves against plate-immobilized Aβ conformers were determined by diluting mAbs with assay buffer into duplicate wells. Antibody binding to blocked wells that had no Aβ, and wells that had Aβ but no primary antibody, served as background controls. Biotinylated goat anti-mouse IgG (γ-chain specific, Sigma-Aldrich) was then applied and detected using streptavidin-horse radish peroxidase (Jackson ImmunoResearch Laboratories, Inc.) and 3,3′,5,5′—tetramethylbenzidine (TMB) substrate (SureBlue Reserve™; KPL, Gaithersburg, MD, USA). Antibody binding curves were generated by subtracting background from assay signal, and the resultant graphs were fitted using a standard 3-parameter sigmoid (logistic) function (SigmaPlot 2000, version 6; Systat Software, Chicago, IL). The concentration of antibody that gave half-maximal binding, EC_50_, and maximum signal amplitude were determined from the fitted curves.

Competition ELISA was performed using microtiter plate wells coated with 200 ng of Aβ monomers and subsequently blocked. Solution-based Aβ conformers were serially diluted (0–0.1 mg/ml) into the coated wells. Then, antibody was immediately added to each well at a concentration equal to twice its EC_50_ value determined by direct ELISA, and the ability of each Aβ conformer to inhibit antibody binding to plate-immobilized Aβ was determined. Background control wells included blocked wells that had no Aβ, and wells that had Aβ but no primary antibody added. The competitor concentration that produced half-maximal inhibition of antibody binding, IC_50_, was determined from sigmoidally fitted curves.

Capture ELISA was performed to assess an mAb’s ability to capture Aβ conformers in solution using plate-immobilized antibodies (200 ng per well). Briefly, synthetic Aβ conformers were serially diluted (0–10 μg/ml) with assay buffer into appropriate microtiter wells. The amount of antibody-bound to Aβ was determined using a polyclonal rabbit anti-Aβ antibody, AW7^71^, a HRP-conjugated donkey anti-rabbit IgG (whole molecule, GE Heathcare, Buckinghamshire, UK) and TMB substrate (SureBlue Reserve™; KPL). EC_50_ values and maximum signal amplitudes were determined from sigmoidally fitted Aβ binding curves.

Surface plasmon resonance (SPR) was used to assess antibody-Aβ binding studies using a Biacore 3000 optical biosensor (Piscataway, NJ) at room temperature and running buffer consisting of PBS containing 0.05% tween 20, pH 7.4. CM5 Chips (Biacore, Uppsala, Sweden) were activated using *N*-ethyl-*N*’-(dimethylaminopropyl)cabodiimide (EDC) and *N*-hydroxysuccinimide (NHS) (GE Healthcare) and IgG (3648 ± 658 response units (RU)) or PFs (3344 ± 290 RU) conjugated to chips via primary amines in optimized immobilization buffer (10 mM sodium acetate pH 4.0–5.5). Reference flow cells consisted of activated chip surfaces that were blocked with ethanolamine. Each binding experiment consisted of analyte mAb (0–1 μM), Fab (0–1 μM), or an Aβ conformer (0–1 μM) flowed over a chip at 30 μL/min. Sensograms were recorded with association and dissociation phases monitored for 300 s and 600 s, respectively. Control studies confirmed that chip regeneration with 10 mM glycine HCl, pH 2.0–3.0, did not modulate analyte binding. Except for PFs, equilibrium and/or kinetic constants for analyte binding were determined by fitting the sensograms, corrected for reference cell signal, to a simple 1:1 Langmuir binding model or to steady state analysis using BIAevaluation software (version 3.2, Biacore Inc.). IgG binding parameters were not determined for experiments involving PFs analyte since binding was essentially irreversible and these assemblies are heterogeneous in size, and presumably, each assembly has a unique propensity for multivalent antibody binding.

### Addition of AD brain extract to iNs and live-cell imaging

Production and characterization of human brain extracts and induced neurons (iNs) from human induced pluripotent cells (iPSCs) are described in the Supplementary Methods. Aliquots (two, 0.5 ml) of mock-immunodepleted (AD) or AW7-immunodepleted brain (ID-AD) extracts were thawed on ice for 30–60 min, vortexed, centrifuged at 16,000 × *g* for 2 min, and buffer exchanged into neurobasal medium supplemented with B27/Glutamax using HiTrap 5 ml desalting column (GE Healthcare, Milwaukee, WI). AD and ID-AD extracts (1 ml) were applied to a desalting column using a 1 ml syringe at a flow rate of ~1 ml/min and eluted with culture medium using a peristaltic pump. In total 10, 0.5 ml fractions were collected. Prior experimentation revealed that the bulk of Aβ eluted in fractions 4 and 5. These two fractions were pooled—this pool is referred to as exchanged extract. A small portion (50 μl) of the exchanged extract was taken for Aβ analysis and the reminder used in iN experiments.

Approximately 7 h prior to exchanging AD and ID-AD extracts into culture medium, iN day 21 neurons were placed in an IncuCyte Zoom live-cell imaging instrument (Essen Bioscience, Ann Arbor, MI). Four fields per well of a 96 well plate were imaged every 2 h for a total of 6 h. This analysis was used to define neurite length and branch points prior to addition of brain extracts. Buffer exchanged brain extracts were diluted 1:2 with culture medium. Half of the medium on iNs was removed (~100 μl) and replaced with 100 μl of 1:2 diluted buffer-exchanged extract – yielding a 1:4 diluted extract on iNs. Similarly, treatments using 1:8 and 1:16 diluted extracts were done in a similar manner. For long-term, continuous imaging, images of four fields per well were acquired every 2 h for 3 days (starting at iN day 21). Whole image sets were analyzed using Incucyte Zoom 2016A Software (Essen Bioscience, Ann Arbor, MI). The analysis job Neural Track was used to automatically define neurite processes and cell bodies based on phase contrast images. Typical settings were: Segmentation Mode—Brightness; Segmentation Adjustment—1.2; Cell body cluster filter—minimum 500 μm^2^; Neurite Filtering—Best; Neurite sensitivity—0.4; Neurite Width—2 μm. Total neurite length (in millimeters) and number of branch points were quantified and normalized to the average value measured during the 6 h period prior to sample addition. Total neurite length is the summed length of neurites that extend from cell bodies, and number of branch points is the number of intersections of the neurites in image field.

For experiments involving addition of mAbs to iNs, 4 × stocks of mAbs (0.4 to 12 μg/ml) were prepared in iN medium. Half of the medium on iNs was removed (~ 100 μl) and replaced with 50 μl of mAb stock plus either 50 μl exchanged extract or 50 μl fresh iN media. Thus, the mAb concentrations tested ranged from 0.1 to 3 μg/ml and were applied in the presence and absence of 1:4 diluted AD brain extract.

### Data analysis and Statistical test

Experiments shown in all Figures data are representative of at least 2 independent experiments. For live-cell imaging experiments, samples and treatments were coded and tested in a blinded manner. Differences between groups were tested with two-way analysis of variance (ANOVA) with Bonferroni post-hoc tests or student’s *t*-tests (^#^*p* < 0.05, ^##^*p* < 0.01, and ^###^*p* < 0.001).

### Data availability

All data generated or analyzed during this study are included in this published article (and its supplementary information files) and all raw data are available from the authors upon reasonable request.

## Electronic supplementary material

Supplementary Information
