# Supplementary material for: An in vitro paradigm to assess potential anti-Aβ antibodies for Alzheimer’s disease
Source: Nat Commun. 2018 Jul 11;9:2676. doi: 10.1038/s41467-018-05068-w (PMC6041266; doi:10.1038/s41467-018-05068-w)

Supplementary Materials

Supplementary Methods

Preparation of amyloidogenic conformers

Aβ(1-40) monomers, disulfide cross-linked S26C Aβ dimers, $[Aβ(1–40)S26C]_2$ and protofibrils formed from $[Aβ(1–40)S26C]_2$ (PFs) were prepared and characterized as described previously[49,50]. To generate highly pure monomers, 1 mg lots of Aβ(1–40) or Aβ(1–40)S26C were dissolved in 0.5 ml of 7 M guanidine hydrochloride containing 5 mM βME and chromatographed on a Superdex 75 10/300 column (GE Healthcare) eluted in 50 mM ammonium bicarbonate, pH 8.5. Peak fractions were collected and pooled, and peptide concentration determined using the molar extinction coefficient for tyrosine ($\varepsilon 275$ = 1400 M^{-1} cm^{-1}). SEC-isolated Aβ(1–40) was diluted to ~0.2 mg/ml in ice-cold 25 mM ammonium acetate, pH 8.5, aliquoted, frozen on dry ice, and then stored at -80°C. To form $[Aβ(1–40)S26C]_2$, reduced Aβ(1–40)S26C monomer was diluted to 40 μM and incubated at room temperature (RT) and bubbled with oxygen for 5 minutes every 24 hours for 72 hours[49]. Following crosslinking, the reaction mixtures were freeze-dried. Lyophilized peptides were re-dissolved in 3 ml of 7 M guanidine hydrochloride and incubated overnight at RT and the Aβ dimer was isolated using a Superdex 75 16/60 column eluted in 50 mM ammonium bicarbonate, pH 8.5. Peak fractions of dimer were pooled and their concentration determined by absorbance at 275 nm using the molar extinction coefficient for tyrosine ($\varepsilon 275$ = 1400 M^{-1} cm^{-1}) and taking account of the fact that dimers contain 2 tyrosine residues. Meta-stable $[Aβ1\text{-}40S26C]_2$ PFs were generated by incubating $[Aβ(1–40)S26C]_2$ in 20 mM sodium phosphate, pH

7.4, at 37°C for 3 days[49,50]. Aβ monomers and PFs were stored in working aliquots at -80°C. Immediately prior to antibody binding studies, Aβ conformers were thawed and then centrifuged at 16,000 g and 4°C for 20 minutes, and the supernatants were used.

Generation and selection of the novel murine anti-Aβ mAb 1C22

The covalently stabilized synthetic Aβ dimer, $[A\beta 1\text{-}40S26C]_2$, readily assemblies to form kinetically trapped protofibrils (PFs)[49,50] and as such provides a convenient form of assembled Aβ (free of both Aβ monomer and fibrils) to use as an immunogen and a tool to identify aggregate-preferring mAbs. Five, 4 month-old BALB/c mice (Charles Rivers Laboratories, Wilmington, MA) and 5, 4 month-old B6D2F1 (Jackson Labs (Bar Harbor, ME) mice were immunized 4 times with ~30 μg/mouse of protofibrillar $[A\beta 1\text{-}40S26C]_2$ emulsified in aluminium and magnesium hydroxide (Imject ALUM™, Pierce, Rockford, IL) and sera collected 3 days after the final immunization. The presence of antibodies capable of recognizing fresh protofibrillar $[A\beta 1\text{-}40S26C]_2$ was then assessed using a direct ELISA in which $[A\beta 1\text{-}40S26C]_2$ PFs were immobilized on the plate and mouse sera then serially diluted across the plate and the amount of antibody bound to the immobilized Aβ determined using a goat anti-mouse IgG (γ specific)-biotin conjugate and streptavidin-HRP. All mice produced at least some anti-Aβ response. Seven animals had titers ≥150,000 and these were used to generate hybridomas. Spleens were harvested and fused with SP20 cells according to published methods. Supernatants from more than 7,000 hybridomas were screened for IgGs capable of binding to immobilized $[A\beta 1\text{-}40S26C]_2$ PFs. Three hundred and seventy-seven positive clones were identified, and of these, 64 retained anti-Aβ reactivity after sub-cloning.

The 31 clones which exhibited the highest levels of binding to [Aβ1-40S26C]$_2$ PFs were grown in 175 cm^2 flasks and 25 of these yielded useful amounts of purified mAbs and were re-analyzed for their ability to bind to immobilized [Aβ1-40S26C]$_2$ PFs. Six mAbs exhibiting EC_{50} ≥1 nM were then analyzed using a 4-tiered screening procedure (Figure S1), the goal of which was to identify mAbs that preferential bind non-monomeric forms of Aβ. The results of this screen identified 1C22, an IgG_1, as the mAb which exhibited the strongest relative preference for [Aβ1-40S26C]$_2$ PFs.

All animal procedures were performed in accordance with the National Institutes of Health Policy on the Use of Animals in Research and were approved by the Harvard Medical School Standing Committee on Animals or under license from the Department of Health, Republic of Ireland with approval by the animal research ethics committee of University College Dublin.

Production of Fab fragments

Fab fragments were generated using an Fab kit (Thermo Fisher Scientific, Waltham, MA) that contained agarose-immobilized ficin. Briefly, each IgG was diluted to 2 mg/ml in digestion buffer containing 25 mM cysteine (Thermo Fisher Scientific, Waltham, MA) and incubated on a spin column containing agarose-immobilized ficin for 5 hours at 37°C. Fab fragments were purified from intact antibodies and Fc fragments using agarose-immobilized protein A-agarose spin columns. Finally, Fab fragments were separated from residual F(ab')$_2$ by size exclusion chromatography using a Superdex 75 10/300GL column (GE Healthcare, Uppsala, Sweden) eluted with PBS, pH 7.4. Non-reducing SDS-PAGE and silver stain detection confirmed that the Fab fragments were >90% pure.

Fab concentration was determined using the molar extinction coefficient $\varepsilon 280 = 210{,}000^{-1}$ cm^{-1}. Thereafter, the activity of purified Fabs was confirmed by ELISA. Plates were coated with PFs, then test and control Fabs added, and these detected using either biotinylated goat anti-mouse IgG (FCγ, Jackson ImmunoResearch Laboratories, Inc, West Grove, PA) or goat anti-mouse Fab specific (Sigma-Aldrich).

Production of induced neurons (iNs) from human induced pluripotent cells (iPSCs)

The YZ1 iPSC line was obtained from UCONN stem cell core and used to prepare neurogenin 2 (Ngn2)-induced human neurons ([34] and Figure 4). iPSCs were maintained in media containing DMEM/F12, Knockout Serum Replacement, pencillin/streptomycin/glutamine, MEM-NEAA, and 2-mercaptoethanol (all from Invitrogen, Carlsbad, CA) with addition of 10 µg/mL bFGF (Millipore, Billerica, MA) directly prior to media application. Neuronal differentiation was performed via a doxycycline induced Neurogenin 2 system ([34], and Figure 4). iPSCs were plated at a density of 95,000 cells/cm^2 for viral infection. Lentiviruses were obtained from Alstem with "ultrapure titres" and used at the following concentrations: pTet-O-NGN2-puro: 0.1 ul/ 50,000 cells; Tet-O-FUW-eGFP: 0.05ul/ 50,000 cells; Fudelta GW-rtTA: 0.11ul/50,000 cells. To induce Neurogenin 2 expression doxycycline is added on "iN day 1" (Figure 4A) at a concentration of 2 µg/ml. On iN day 2 puromycin is added at 10 mg/ml and is maintained in the media at all times thereafter. On iN day 4, cells were plated at 50,000 cells/well on matrigel (BD Biosciences, San Jose, CA)-coated Greiner 96 well microclear plates and maintained in media consisting of Neurobasal medium (Gibco), Glutamax, 20% Dextrose, MEM NEAA with B27, with BDNF, CNTF, GDNF (PeprpTech, Rocky Hill, NJ) each at a concentration of 10 ng/ml. At designated time points, iNs were characterized using live-cell imaging to monitor neuritic

complexity (Figure 4A-C), and used for immunocytochemistry (Figure 4D) or Western blotting to assess the expression of neuronal markers (Figure S4). At iN day 14 neurite number and expression of neural markers had reached near maximal levels. Thus, for experiments investigating the effects of AD brain extracts on neuronal viability iNs were used at iN day 21, a time point when iNs were fully mature.

Western blot and immunocytochemical characterization of induced neurons (iNs) Cells grown in 96 well plates were lysed by the addition of 50 µl lysis buffer (0.1% SDS, 1% NP-40, 50 mM HEPES, pH 7.4, 2 mM EDTA, 100 mM NaCl, 5 mM Na_3VO_4, 40 µM p-nitrophenyl phosphate, plus protease inhibitors) and incubated at 4°C for 30 minutes. Lysates were centrifuged at 15,000 g and 4°C for 25 minutes in a bench top Eppendorf centrifuge. Supernatants were collected and protein content determined using a BCA kit (Thermo Fisher Scientific, Waltham, MA). Twenty µg of total protein was loaded in each lane and electrophoresed on pre-cast 15 well 4-12% polyacrylamide Bis-Tris LDS gels (Invitrogen, Carlsbad, CA). Proteins were transferred onto 0.2 µm nitrocellulose at 400 mA for 2 hours and blots incubated overnight at 4°C with primary antibody (Table S1). Membranes were washed three times for 10 minutes with PBST and then incubated in PBS containing 0.02%(w/v) SDS for 1 hour with either goat anti-mouse infrared 800 antibody (Rockland, Gilbertville, PA) diluted 1:15,000 or goat anti-rabbit infrared 700 antibody (Rockland, Gilbertville, PA) diluted 1:5,000 for 1 hour at RT. Membranes were washed three times for 10 minutes with PBST and then a further two times with PBS and immunoreactive bands were visualized using a Li-COR Odyssey infrared imaging system (Li-COR, Lincoln, NE).

The maturity of DIV21 iNs was also assessed using immunocytochemistry and confocal microscopy. Cells were fixed in 4% paraformaldehyde (PFA) and 4% sucrose at room temperature for 15 minutes, and then permeabilized with ice-cold methanol for 3 minutes. Cells were then washed with PBS 3 times and blocked using 5%(w/v) BSA in PBS containing 0.02% sodium azide. Thereafter, iNs were incubated overnight with primary antibodies (Table 1) at 4^oC. Cells were again washed 3 times with PBS and then incubated for 1 hour at room temperature with fluorescence-conjugated secondary antibodies (AlexaFluor 546 goat anti-mouse; and AlexaFluor 633 goat anti-rabbit). Finally, iNs were washed 3 times with PBS and examined using a Zeiss LSM710 confocal microscope fitted with a 40x air objective (NA: 0.8). Images were captured in a Z-stack manner (5-10 stacks, interval 0.5-1.0 μm) and maximal pixel intensity projections were created with averaging of 2 frames set to 1024 x 1024 pixel resolution.

Preparation of human brain extracts

Human tissue was used in accordance with the Partner's Institutional Review Board (Protocol: Walsh BWH 2011). Frozen hemibrains from 2 end-stage AD cases (referred to as AD1 and AD2) were obtained from the Massachusetts ADRC Neuropathology Core, Massachusetts General Hospital). AD1 was a 68 years old female with end-stage AD and fulminant amyloid and neurofibrillary tangle pathology. AD2 was a 69 year old woman who had pathological changes consistent with end-stage AD. Approximately 20 g of cortical gray matter was dissected from each case and this material was then sliced into ~2 g lots with a razor blade and homogenized in artificial cerebrospinal fluid base buffer (aCSF-B) (124 mM NaCl, 2.8 mM KCl, 1.25 mM NaH_2PO_4, 26 mM $NaHCO_3$, pH 7.4). aCSF-B was supplemented with protease inhibitors (5 mM

ethylenediaminetetraacetic acid, 1 mM ethyleneglycoltetraacetic acid, 5 µg/ml Leupeptin, 5 µg/ml Aprotinin, 2 µg/ml Pepstatin, 120 µg/ml Pefabloc and 5 mM NaF) and tissue homogenized in 5 volumes of ice-cold aCSF-B with 25 strokes of a Dounce homogenizer (Fisher, Ottawa, Canada). Resulting 20% (w/v) homogenates were centrifuged at 200,000 g for 110 minutes and 4°C in a SW41 Ti rotor (Beckman Coulter, Fullerton, CA). The upper 80% of the supernatant was removed and dialyzed against fresh aCSF-B to eliminate small molecules and drugs. A total of 50 ml of clarified homogenate was dialyzed (using Slide-A-Lyzer™ G2 Dialysis Cassettes, 2K MWCO, Fisher Scientific) at 4°C against a 100-fold excess of aCSF-B with buffer changed 3 times over a 72 hour period. Thereafter, the dialysate was divided into 2. One portion was immunodepleted (ID) of Aβ by 3 rounds of 12 hour incubations with the anti-Aβ antibody, AW7, plus Protein A sepharose (PAS) beads at 4 °C. The second portion was treated in an identical manner, but incubated with pre-immune rabbit serum plus PAS beads. Samples were cleared of beads and 0.5 ml aliquots stored at -80°C until used for biochemical or IncuCyte experiments. Samples were thawed once and used.

It is possible the manipulations necessary to process tissue may lead to changes in the aggregation state and dynamics of brain-derived Aβ, but this is unavoidable. However, to account for changes that might occur during immunodepletion we included an identical sample that was "mock immunodepleted" using pre-immune serum, and freeze thawing was avoided by aliquotting samples immediately after their preparation and using samples (mock immunodepleted, immunodepleted and control brain) only once.

Immunoprecipitation/Western blot analysis of amyloid β-protein

Extracts were first pre-cleared with PAS beads to minimize non-specific interactions in the subsequent IP. Half milliliter aliquots of extracts were incubated with 15 µl PAS beads for 1 hour at 4°C with gentle shaking. PAS beads were removed by centrifugation (4,000 g for 5 minutes), the supernatant removed and incubated with 10 µl of AW7 and 15 µl PAS beads overnight at 4°C with gentle shaking. Aβ-antibody-PAS complexes were collected by centrifugation and washed as previously described[27]. The immunoprecipitated (IP'd) Aβ was eluted by boiling in 15 µl of 2× sample buffer (50 mM Tris, 2% w/v SDS, 12% v/v glycerol with 0.01% phenol red) and electrophoresed on hand poured, 15 well 16% polyacrylamide tris-tricine gels. Western blotting and immunodevelopment were done essentially as described above. Synthetic Aβ1-42 was run as a loading control and protein transferred onto 0.2 µM nitrocellulose at 400 mA and 4°C for 2 hours. Blots were microwaved in PBS and Aβ detected using the anti-Aβ40 and anti-Aβ42 antibodies, 2G3 and 21F12, and bands visualized using a Li-COR Odyssey infrared imaging system (Li-COR, Lincoln, NE).

MSD Aβ immunoassay

Samples were analyzed for Aβ content using an assay specific for Aβx-42, which preferentially detects Aβ monomers[27,70]. This assay was performed using the Meso Scale Discovery (MSD) platform and reagents from Meso Scale (Rockville, MD). m266 (3 µg/ml) was used for capture and biotinylated 21F12 (1 µg/ml) for detection. Samples, standards and blanks were loaded in triplicate and analyzed as described previously[27,70].

Since GuHCl effectively disaggregates high molecular weight Aβ species[27,34], samples were analyzed both with and without incubation in 3.5 M GuHCl. Analysis of samples in the absence of GuHCl allows the measurement of native Aβ monomer, whereas, analysis of samples treated with GuHCl allows detection of disassembled aggregates with the Aβx-42 assay. To dissociate aggregates 20 µl of extract was incubated overnight with 20 µl of 7 M GuHCl at 4°C. Thereafter, samples were diluted 1:7 with assay diluent, so that the final GuHCl concentration was 0.5 M. Aβ standards were prepared in tris-buffered saline, pH 7.4 containing 0.5 M GuHCl, 0.05% Tween 20 and 1% Blocker A so that both standards and samples contained the same final concentration of GuHCl.

Human immunostaining

The brain was collected from an 82 year old man (PMI = 8 hours) who died with end-stage Alzheimer's disease and 8 μM thick serial cryosections were prepared from the inferior temporal gyrus (Brodmann area 20). Cryosections were mounted on Superfrost+ microscopy slides, fixed with 75% ethanol for 2 minutes and then washed for five minutes with PBS. Sections were then incubated with anti-Aβ antibodies at 4.0 μg/ml in PBS for 40 minutes at 37° C. Sections were washed two times with PBS for 5 minutes each, and then incubated with ImmPRESS™ anti-mouse IgG (Vector Laboratories product MP-2400, Burlingame, CA) secondary antibody for 40 minutes at 37°C. Sections were washed again two times with PBS for 5 minutes each, and then developed with DAB solution (Vector Laboratories product SK-4100) for precisely 4 minutes. Slides were washed again once with PBS for 5 minutes and once with water for 2 minutes, and then dehydrated and cleared through a graded ethanol series into xylene and cover-slipped with paramount.

Photomicroscopy was performed on an Olympus BX40 microscope using visible light. Immunostained slides were photographed at 10x with an Olympus Qcolor camera using Qphoto software with exposure settings held constant and identical throughout.

Supplementary Table 1. MAb binding to PFs and Aβ monomers

Assay	Surface bound		Units	Potential Cons	Antibody-Aβ					
					1C22 (Aβ40 epitope)		3D6 (Aβ1-5 epitope)		266 (Aβ16-23 epitope)	
	Aβ	mAb			PFs	Mon	PFs	Mon	PFs	Mon
Direct ELISA	Yes	No	EC_{50} (pM)	Surface-modulation of Aβ/avidity effects.	5.75 ± 0.01	24.0 ± 0.11	44.7 ± 0.08	18.6 ± 0.03	424.0 ± 42	26.3 ± 0.03
Comp ELISA	Yes	No	IC_{50} (μg/ml	Indirect measure of mAb-Aβ Interactions	1.8 ± 0.03	42.0 ± 0.78	0.04 ± 0.001	1.2 ± 0.02	0.14 ± 0.003	0.06 ± 0.001
Capture ELISA	No	Yes	EC_{50} (μg/ml)	Avidity effects	17.0 ± 7.9	3082 ± 1537	3.7 ± 0.02	4.8 ± 1.2	4.2 ± 2.6	5.1 ± 4.5
SPR	No	Yes	K_D *or* K_{APP} (nM)	Avidity effects	<1 nM	1100 ± 500	<1 nM	7.9 ± 0.16	<1 nM	2.1 ± 1.8

Values for EC_{50}s and IC_{50}s were calculated from antibody binding, capture, and Aβ competition curves as shown in Figures 1 and 2. Values for dissociation constants (K_D) and apparent K_Ds (K_{APP}) were determined from sensograms that were globally fit to a 1:1 langmuir binding model or by steady state analysis of the binding data as shown in Figure 2. Values are means ± SD, with the number of replicates ≥3.

Supplementary Table 2. Primary and secondary antibodies

Antibody	Type	Antigen/epitope	Dilution for IP	Conc. for WB	Conc. for ELISA	Conc. for ICC	Source/Reference
3D6	Monoclonal	Aβ1–5	–	-			Elan/(Johnson-Wood et al., 1997)
6E10	Monoclonal	Aβ3-8	-	1 μg/ml	-		Biolegend/(Kim et al. 1988)
266	Monoclonal	Aβ16-23	–	-	3 μg/ml		Elan/(Seubert et al., 1992)
2G3	Monoclonal	Aβ40	–	1 μg/ml	–		Elan/(Johnson-Wood et al., 1997)
21F12	Monoclonal	Aβ42	–	1 μg/ml	1 μg/ml		Elan/(Johnson-Wood et al., 1997)
1C22	Monoclonal	Aβ aggregates	–	–	-		Walsh lab/(Mably et al., 2015, Yang et al. 2015)
AW7	Polyclonal	Pan anti-Aβ	1:80	–	–		Walsh lab/(Mc Donald et al., 2012)
46-4	Monoclonal	HIV glycoprotein 120	-	-	-	-	ATCC# CRL-2178/(Reeves et al., 1995)
αGluA1	Monoclonal	AMPA-R subunit GluA1	-	1 μg/ml	-	-	Millipore (04-855)
αPSD-95	Monoclonal	Post-synaptic density protein 95	-	1 μg/ml	-	-	Millipore (MAB1596)
αSYN1	Polyclonal	Synapsin 1	-	1 μg/ml	-	-	Millipore (AB1543)
αSYT	Monoclonal	Synaptophysin	-	1 μg/ml	-	-	Millipore (MAB5528)
K9JA	Polyclonal	Tau (243-441)	-	1:1,000	-	1:1000	DAKO (A0024)
AT8	Monoclonal	pS202/pS205				1:200	Thermo Scientific (MN1020)
αMAP2	Monoclonal		-	-	-	1:500	Millipore (MAB3418)
αNeuN	Monoclonal		-	-	-	1:500	Millipore (MAB377)
αGAPDH	Monoclonal		-	1 μg/ml	-	-	Millipore (CB1001)
αRb-IR tag	Polyclonal	Rabbit IgG	-	1:5,000	-	-	LiCor (925-68071)
αMse-IR tag	Polyclonal	Mouse IgG	-	1:5,000	-	-	LiCor (925-32210)
αRb-Alex546	Polyclonal	Rabbit IgG	-	-	-	1:1,000	Thermo Scientific (A11030)
αMse-Alex633	Polyclonal	Mouse IgG	-	-	-	1:1,000	Thermo Scientific (A21070)

Supplementary Figures Legends and Figures

Supplementary Figure 1. Production and characterization of Aβ monomers, dimers and protofibrils. (**A**) Aβ1-40 and (**B**) $[\text{A}\beta\text{1-40S26C}]_2$ peptides were incubated overnight at 2 mg/ml in 7 M guanidine hydrochloride and monomers and dimers isolated using a HiLoad 16/60 Superdex 75 column eluted with 50 mM ammonium acetate, pH 8.5. **(C, D** and **E)** After incubation in 20 mM sodium phosphate buffer for 3 days $[\text{A}\beta\text{1-40S26C}]_2$ assembled to form thioflavin T positive PFs which eluted in the void of a Superdex 75 10/300 column. The size bar on the micrograph is 100 nm.

Jin, O'Nuallain *et al.*, Supplementary Figure 1

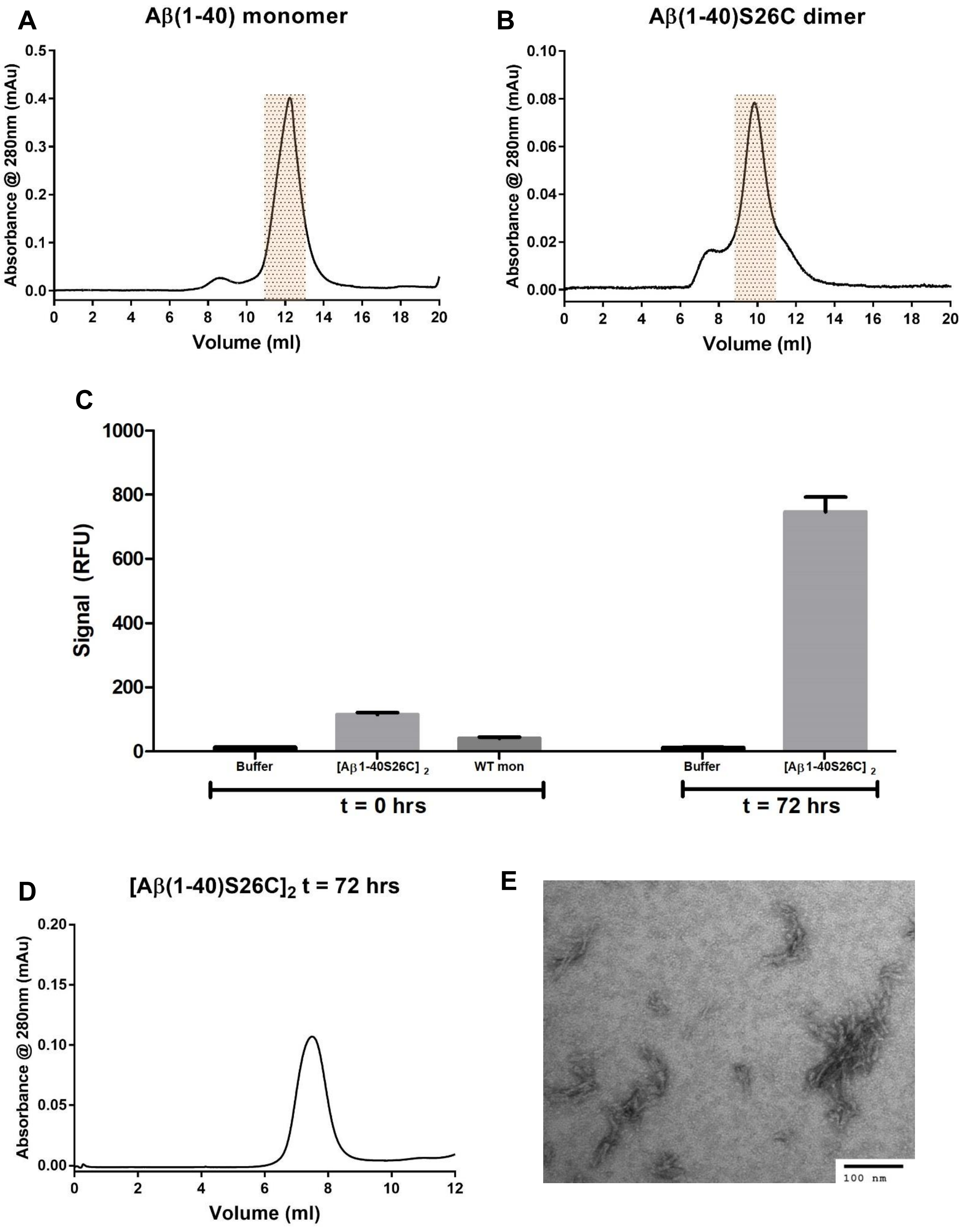

Supplementary Figure 2. Schematic describes the 4 stage screening process used to select 1C22 from an initial pool of ~7,000 hybridoma. A direct ELISA which utilized plate-immobilized [Aβ1-40S26C]$_2$ PFs (1st screen) identified positive hybridomas and subsequent subclones. Thirty one clones which showed the strongest reactivity with [Aβ1-40S26C]$_2$ PFs were then tested for binding to plate-immobilized monomer (2nd screen), solution-phase monomer and solution-phase [Aβ1-40S26C]$_2$ PFs (3rd screen and 4th screen).

Jin, O'Nuallain *et al*., Supplementary Figure 2

1st screen: Protofibril ELISA

Protofibrils formed from $[A\beta 1\text{-}40S26C]_2$ were immobilized on the surface of 96 well plates and each purified mAb was serially diluted across the plate and the conc. of mAb that produced 50% maximal binding (EC_{50}) was determined from fitted dilution curves.

↓

2nd screen: Monomer ELISA

$A\beta$ monomer was isolated using SEC and immediately immobilized on the surface of 96 well plates and each purified mAb was serially diluted across the plate and the conc. of mAb that produced 50% maximal binding (EC_{50}) was determined from fitted dilution curves.

↓

3rd screen: competition ELISA

mAbs were tested for their ability to bind to plate-immobilized $A\beta$ monomer in the presence of increasing amounts of solution-phase protofibrils or monomer. The amount of mAb was kept constant and binding of mAb to protofibrils was plotted vs. the conc. of competing conformer. The conc. of conformer that caused a 50% reduction in binding to protofibrils (IC_{50}) was determined.

↓

4th screen: Capture ELISA

mAbs were immobilized on the surface of 96 well plates and freshly isolated monomer, $[A\beta 1\text{-}40S26C]_2$ or $[A\beta 1\text{-}40S26C]_2$ protofibrils diluted across the plate, allowed to bind to the mAb and detected with either a polyclonal anti-$A\beta$ antibody or a mAb antibody to the extreme C-terminus of $A\beta 40$. The conc. of each $A\beta$ conformer that produced 50% maximal binding (EC_{50}) was determined from fitted dilution curves.

Supplementary Figure 3. 1C22 binds stronger to full-length Aβ than overlapping peptide fragments. (**A**) Representative antibody binding curves for 1C22 and the anti-Aβ mAb, 6E10, against plate-immobilized Aβ and overlapping Aβ peptide fragments, respectively. The data symbols represent antibody binding to Aβ peptides that are specified in Panel B. (**B**) A schematic of 1C22's reactivity against plate-immobilized Aβ and overlapping peptide fragments.

Jin, O’Nuallain *et al*., Supplementary Figure 3

A

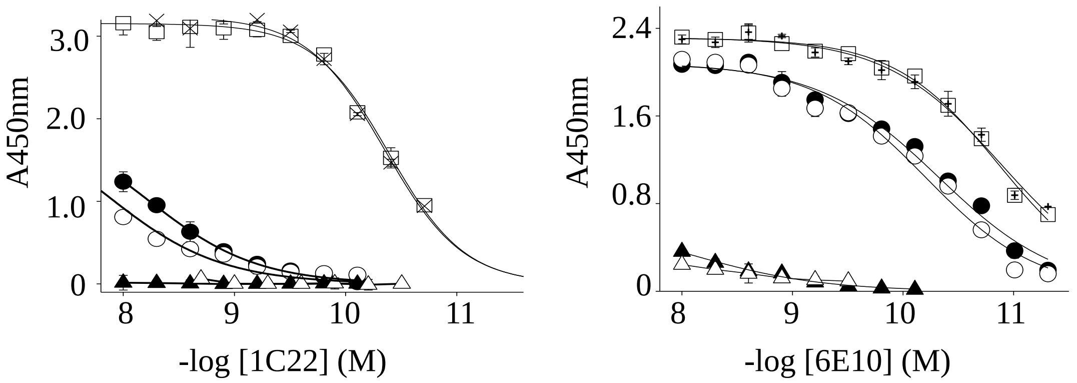

B

Aβ peptide — sequence: **1** DAEFRHDSGY **10** EVHHQKLVFF **20** AEDVGSNKGA **30** IIGLMVGGVV **40**

Aβ peptide	1C22	6E10
● 1-15	+	++++
○ 3-13	+	++++
▲ 11-25	-	-
16-30	-	-
21-35	-	-
26-35	-	-
Δ 17-40	-	-
□ 1-40M	+++	++++
+ S26CM	+++	++++

Supplementary Figure 4. 3D6 readily detects amyloid plaques in fresh frozen AD brain, whereas 1C22 only modestly detects plaques, and 266 minimally recognizes plaques. The recognition of native amyloid plaques by mAbs was determined using simultaneously processed, ordered 8 μm serial sections of human AD cortex were. **(A)** staining with 3D6, **(B)** 1C22, and **(C)** 266. All images were captured at the same magnification and the scale bar in **(A)** is 100 μM. A pair of omnipresent large plaques (arrowheads) is evident throughout these serial sections. Using these two plaques as reference points it is apparent that 3D6 produces the highest binding to plaques, and 266 the least. Staining of plaques by 1C22 is intermediate between that seen with 3D6 and 266. The images shown are representative of analysis of 2 separate experiments.

Jin, O'Nuallain *et al*., Supplementary Figure 4

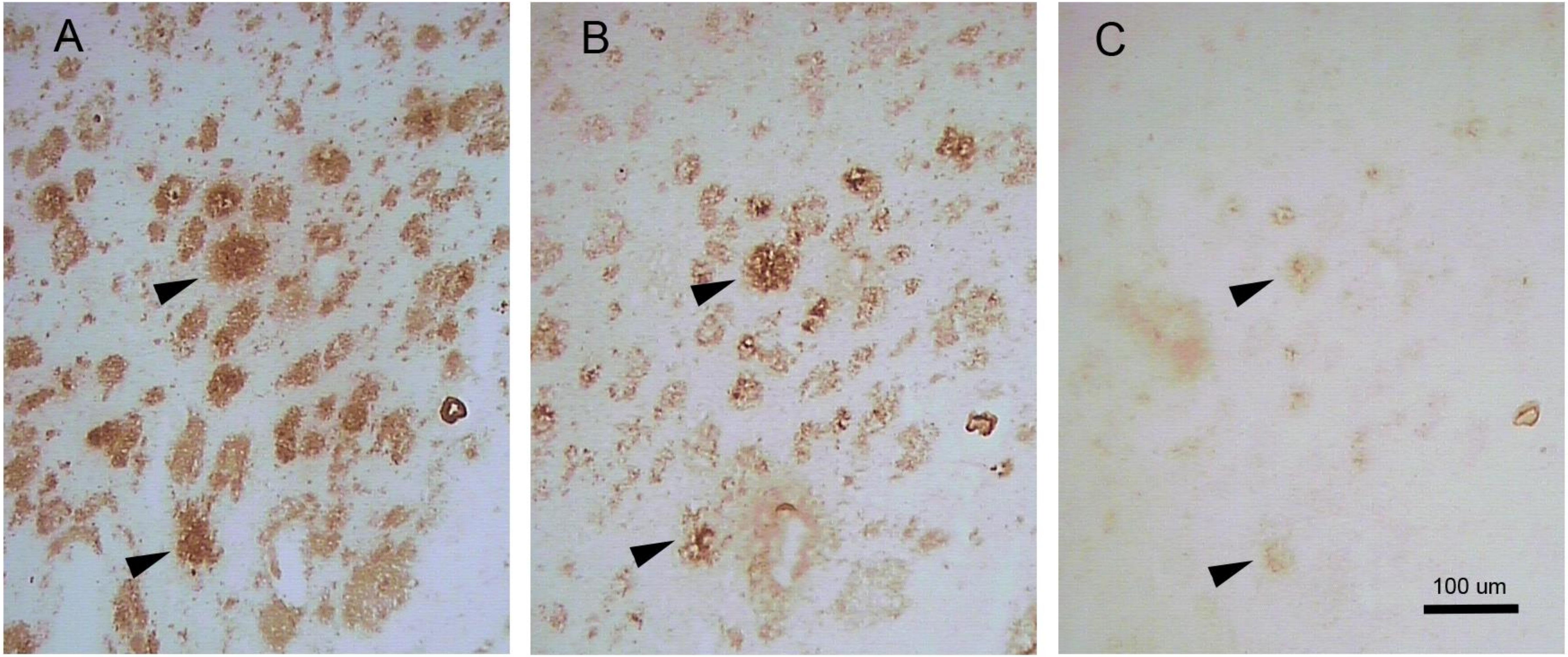

Supplementary Figure 5. Human induced neuron (iNs) have mature neurites and express neural and synaptic markers by post-differentiation day 14. Human induced neuron (iNs) were imaged on iN day 7, 14, 21, 28 and on each of these time points sister wells were lyzed and their contents used for Western blotting. **(A)** The IncuCutye NeuroTrack algorithm was used to quantify neurite length, the number of neurite branch points and the number of cell body clusters. Each data point is the average of measurement from 6 wells of iN cells cultured in the same 96 well plate. Error bars are SEM. **(B)** Equal amounts of protein from lysates collected on iN day 7, 14, 21, and 28 were used for Western blotting employing antibodies to GluA1, the post-synaptic density protein 95 (PSD95), synaptophysin (SYT), synapsin 1 (SYN1), tau (Tau) and the house-keeping enzyme GAPDH. The levels of neuronal markers increased from iN day 14, the same interval at which neurite length and complexity had reached near maximal levels. (**C**) Cultures from iN day 21 days were fixed and stained for the neuronal markers: MAP2, NeuN and Tau, and used for confocal microscopy. The scale bar is 50 µm. Results shown in (**A**)-(**C**) are representative of at least two independent experiments.

Jin, O'Nuallain *et al*., Supplementary Figure 5

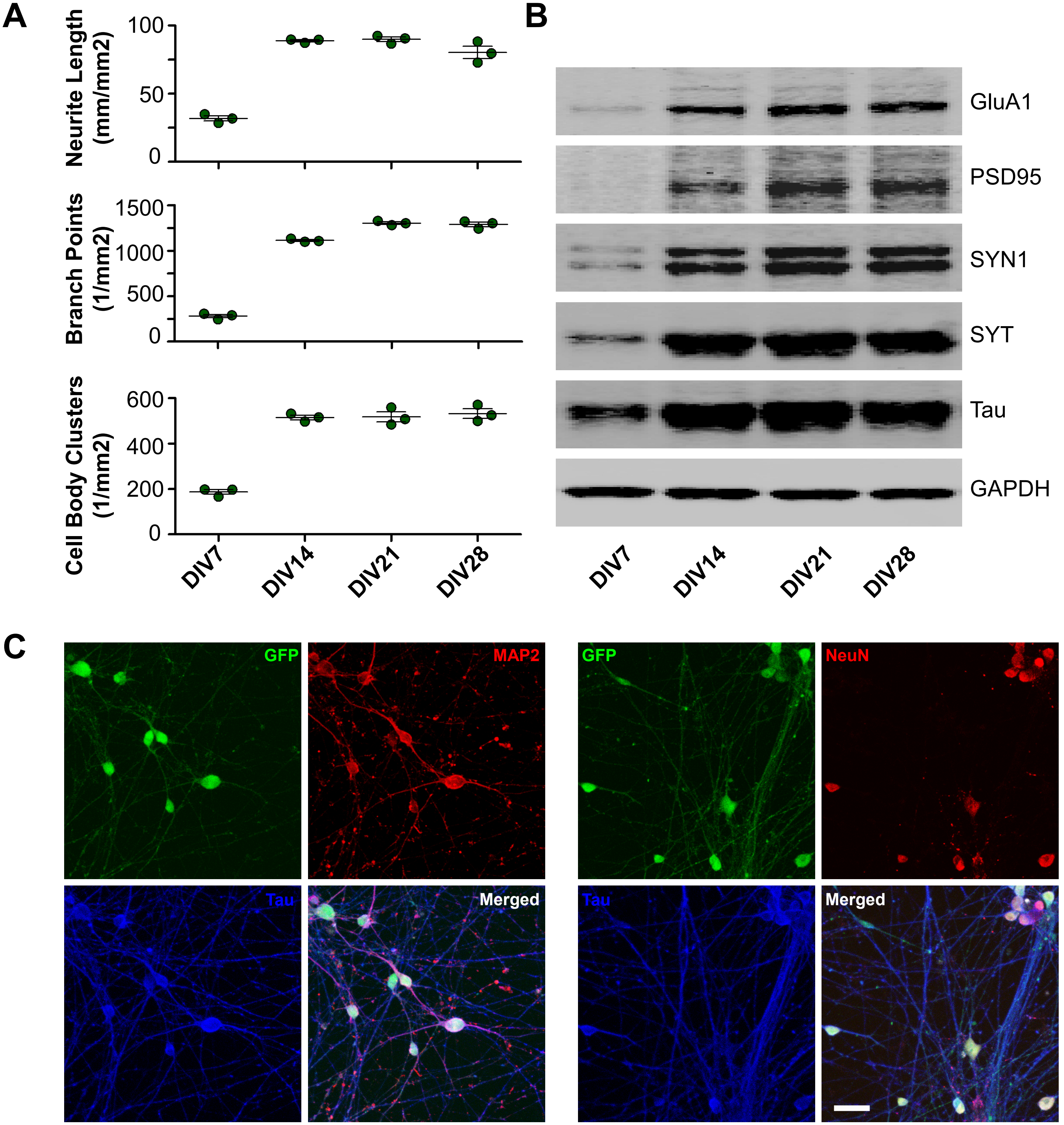

Supplementary Figure 6. Characterization of AD brain extracts used for neuritotoxicity experiments. (A and **D)** Half milliliter aliquots of mock immunodepleted (AD1 and AD2) and AW7 immunodepleted (ID-AD1 and ID-AD2) extracts were analyzed by IP/WB. AW7 was used for IP and a combination of 2G3 and 21F12 was used for WB. To enable comparison between gels 2 ng of Aβ1-42 peptide was also electrophoresed on each gel. IP/WB analysis allows the capture of Aβ structures under native conditions and their detection following denaturing SDS-PAGE. The WB bands detected at ~4 and ~7 kDa is typical of the pattern we have seen in IP/WBs of >100 AD brains and indicates that at least two different Aβ species are present in both the AD1 and AD2 extracts. The same samples were also analyzed (in triplicate) by an MSD-based Aβx-42 immunoassay before **(B** and **E)** and after exchanging into iN growth medium **(C** and **F)**. Results are shown as means ± SD. Since GuHCl effectively disaggregates high molecular weight Aβ species, samples were analyzed with and without incubation in denaturant. Analysis of samples in the absence of GuHCl (-) allows the measurement of native Aβ monomer, whereas, analysis of samples treated with GuHCl (+) allows detection of disassembled aggregates. Both AD1 and AD2 contained much larger amounts of aggregates than monomer, and both monomer and aggregates were effectively removed by AW7 immunodepletion. Exchanging extracts into iN media resulted in ~20-45% loss of Aβ from both the AD1 and AD2 extracts. The experiments shown are typical of at least 3 separate experiments.

Jin, O'Nuallain *et al.*, Supplementary Figure 6

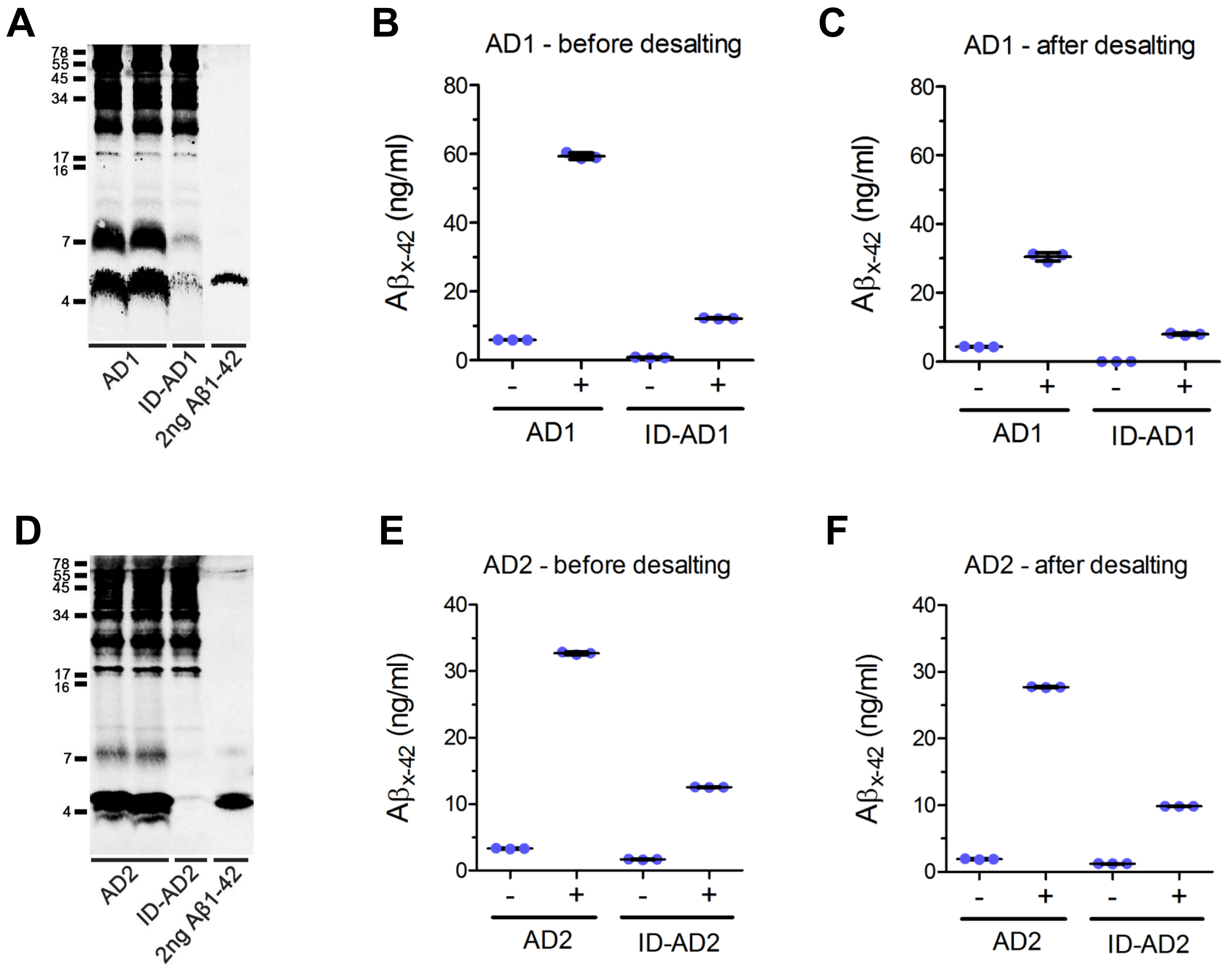

Supplementary Figure 7. Treatment of iNs with AD brain-derived soluble Aβ induces neuritic dystrophy. The raw data used to generate the graphs shown in Figure 5B are graphed to show **(A)** values from individual wells normalized to 6 hour period prior to treatment, and **(B)** umanipulated measures of neurite length and neurite branch points are shown as averages of triplicate wells. In **(A)** each well of iNs was imaged for 6 hours prior to addition of sample and NeuroTrack-identified neurite length and branch points used to normalize neurite length and branch points measured at each interval after addition of sample and the results from individual wells are shown. In **(B)** the same primary data used to generate **(A)** were without normalization to the -6 to 0 hour period, and the averages of results from triplicate wells are shown ± SEM. Results are shown for Mock-ID AD1 extract tested at 2 dilutions, 1:4 and 1:16. Cells treated with medium alone were used to monitor the integrity of untreated cells.

Jin, O'Nuallain et al., Supplementary Figure 7

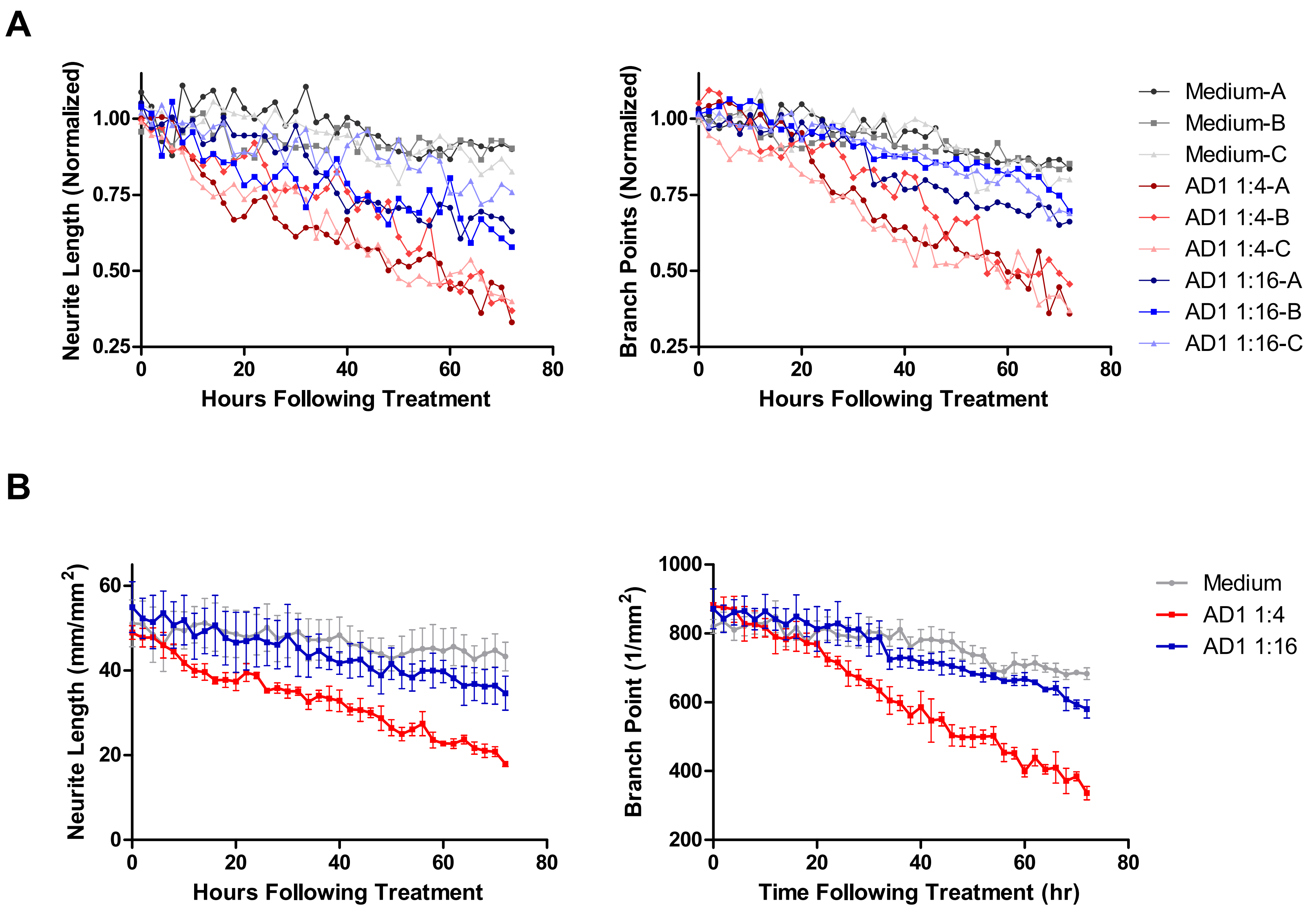

Supplementary Figure 8. Soluble Aβ assemblies from AD brain induced neuritic dystrophy. (A) Human induced neuron (iN day 21) were treated with medium, or AD2 brain extract mock-immunodepleted (Mock ID) or immunodepleted with the anti-Aβ antiserium AW7 (AW7 ID). Phase contrast images (top panels) at 0 and 72 hours were analyzed using the IncuCutye NeuroTrack algorithm to identify neurites (middle panels) and the NeuroTrack-identified neurites (pink) are shown superimposed on the phase contrast image (bottom panels). Scale bars are 100 µm. (**B**) Mock-ID AD2 extract was tested at 3 dilutions, 1:4, 1:8 and 1:16, and immunodepleted AD2 was tested at 1:4. Cells treated with medium alone were used to monitor the integrity of untreated cells. Graphs show the normalized change of neurite length (left panel) and branch points (right panel). Each data point is the average of triplicate wells for each treatment ± SEM. (**C**) The normalized change of cell body clusters was determined for AD1 and AD2 extracts each at a dilution of 1:4. The results shown are representative of at least three independent experiments.

Jin, O'Nuallain *et al.*, Supplementary Figure 8

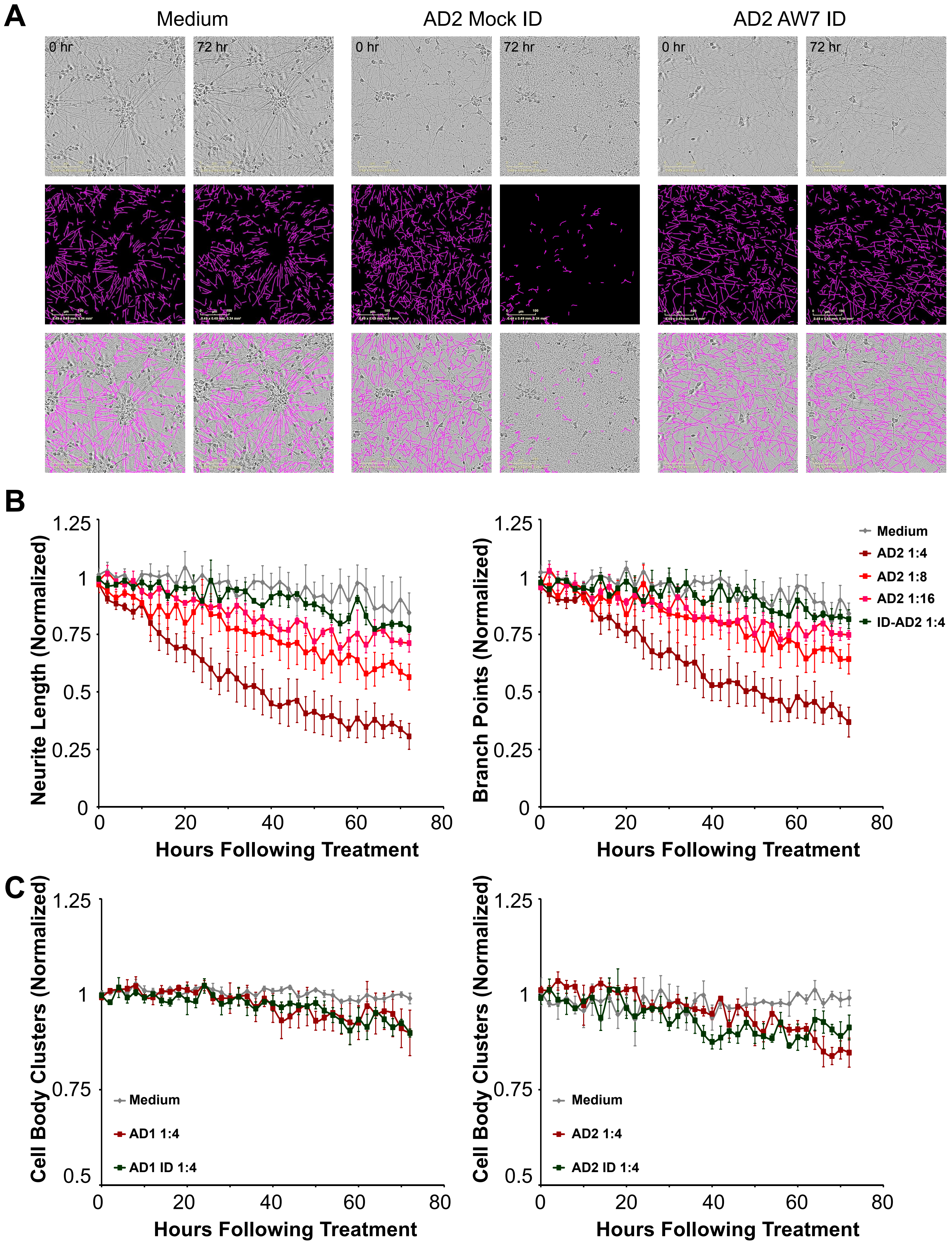

Supplementary Figure 9. Extracts from control brains have no effect on neuronal or neuritic viability. (A) Human induced neuron (iN day 21) were treated with medium, or extract from control brain 1 (cont1) or **(B)** control brain 2 (cont2) at 3 dilutions, 1:4, 1:8 and 1:16. Cells treated with medium alone were used to monitor the integrity of untreated cells. Graphs show the normalized change of neurite length (left panel) and branch points (right panel). Each data point is the average of triplicate wells for each treatment ± SEM. The results shown are representative of at least three independent experiments. Control brain 1 was from an 82 year old male and control brain 2 was from a 58 year old female. Both subjects died free of AD and postmortem evidence failed to find evidence of significant AD pathology and both individuals were assigned a Braak score of II.

Jin, O'Nuallain *et al*., Supplementary Figure 9

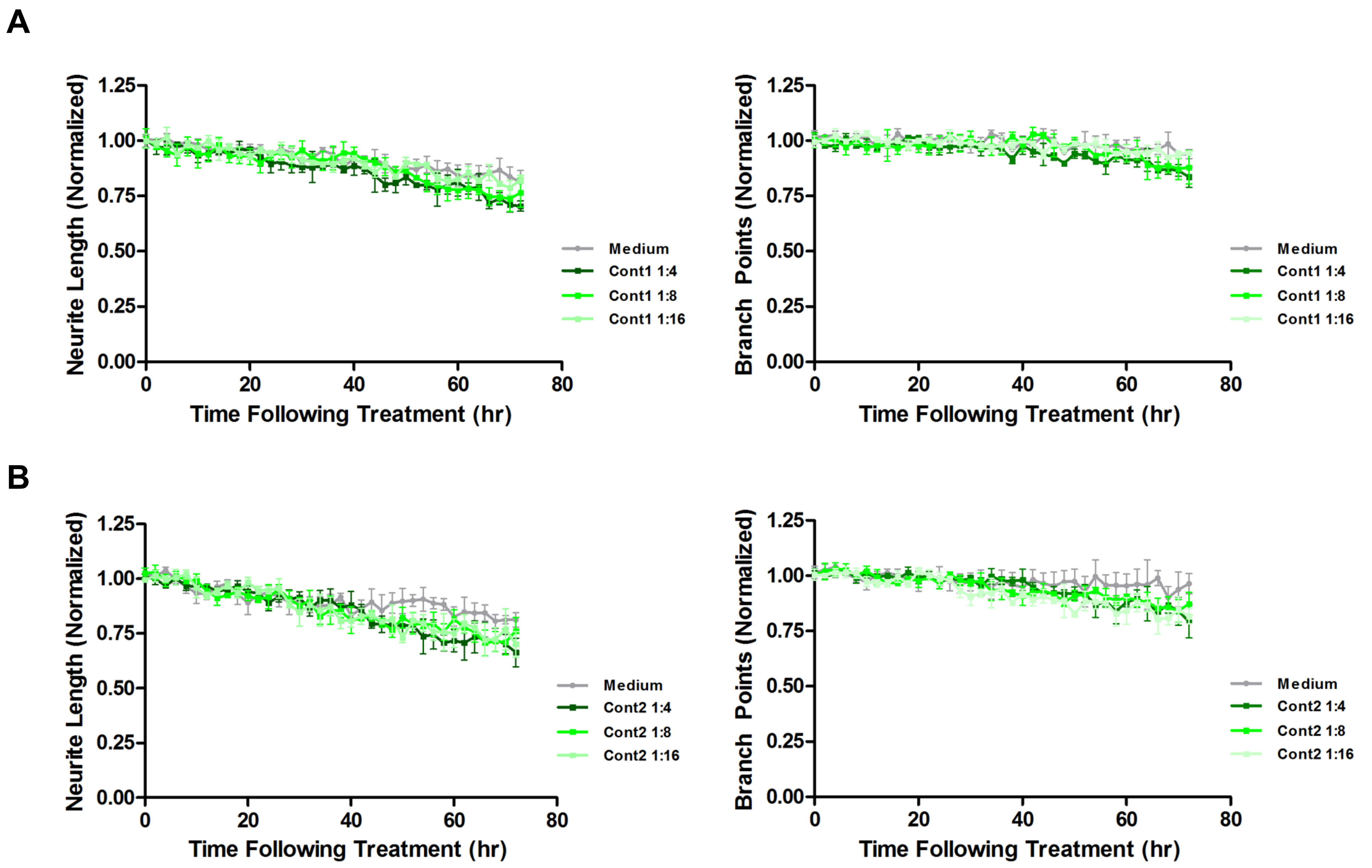

Supplementary Figure 10. Anti-Aβ antibodies dose-dependently attenuate the neuritotoxic effects of AD brain extracts. As in Figure 6, iNs were treated with AD1 extract at a dilution of 1:4 in the presence or absence of increasing amounts of anti-Aβ antibody. Here graphs show time-course measurements of NeuroTrack-defined neurite branch points of iNs treated ± AD1 extract and (**A**) 4-64, (**B**) 266, (**C**) 3D6, and (**D**) 1C22. Each data point is the average of 3 wells ± SEM.

Jin, O'Nuallain *et al.*, Supplementary Figure 10

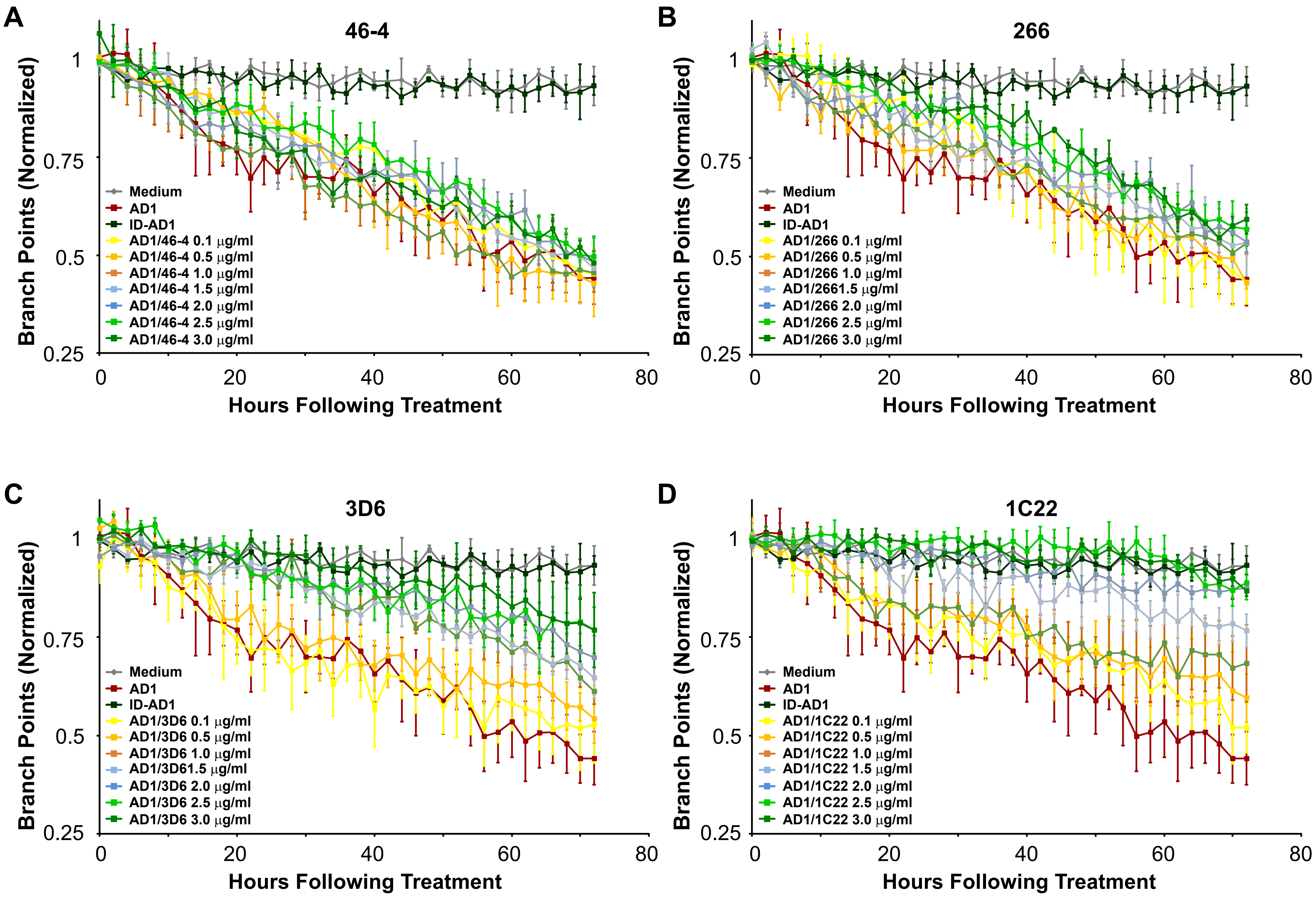

Supplementary Figure 11. Anti-Aβ antibodies dose-dependently attenuate the neuritotoxic effects of an extract from a second AD brain. To investigate whether the relative protective effects of 1C22, 3D6 and 266 were generalizable we investigated whether anti-Aβ antibodies could protect against the neuritotoxicity induced by an extract from a second AD brain, AD2. DIV 21 human induced neuron (iNs) were treated with AD2 extract at a dilution of 1:4 in the presence or absence of increasing amounts of anti-Aβ antibody. Graphs show time-course measurements of NeuroTrack-defined neurite length of iNs treated ± AD1 extract and (**A**) 46-4, (**B**) 266, (**C**) 3D6, and (**D**) 1C22. Each data point is the average of 3 wells ± SEM.

Jin, O'Nuallain *et al.*, Supplementary Figure 11

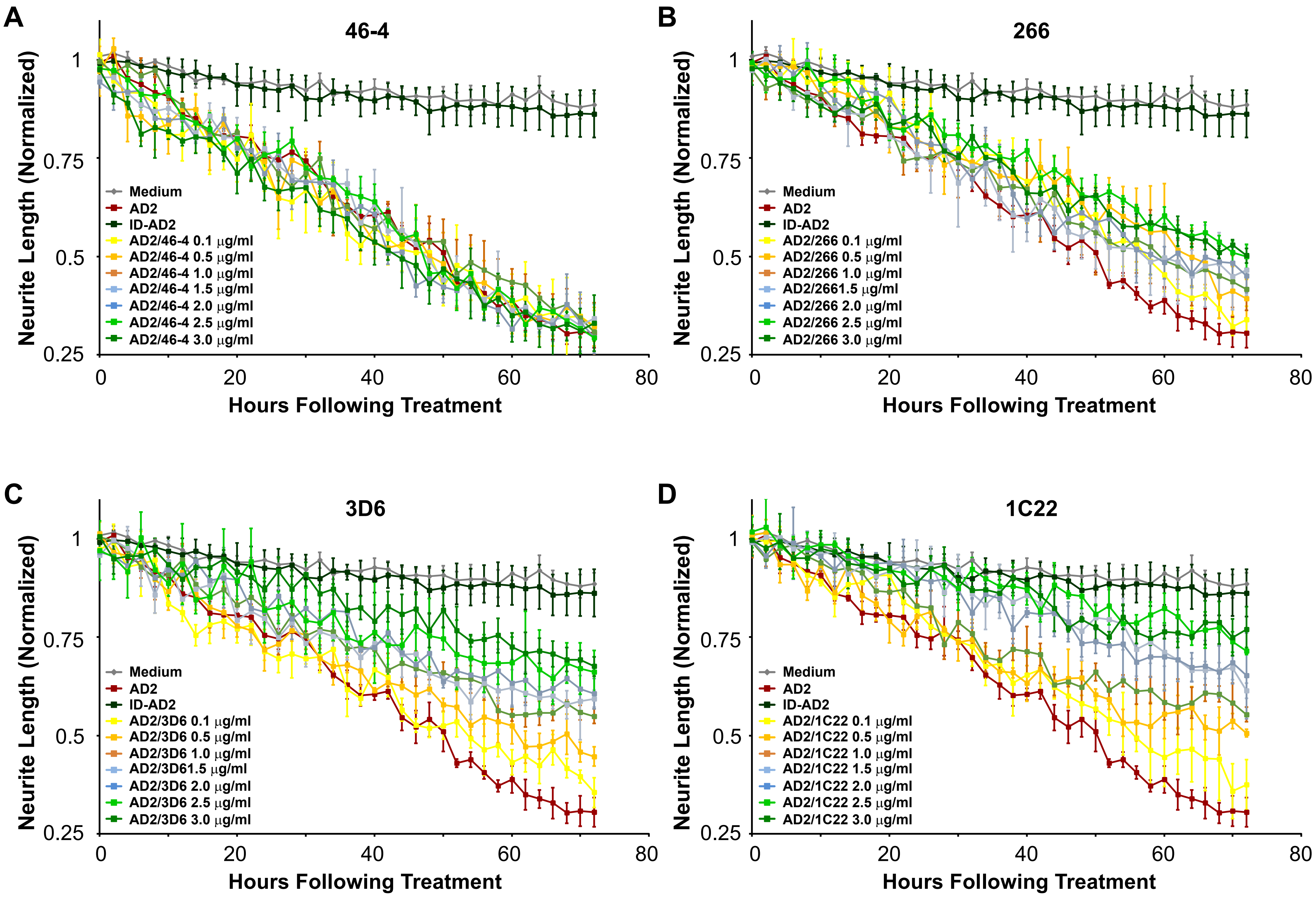

Supplementary Figure 12. Anti-Aβ mAbs attenuate Aβ-induced tau phosphorylation and neuritotoxicity. (A) End-point cultures from the experiment shown in Figure 6 were fixed and stained for phosphorylated tau using the pS202/pS205 specific mAb, AT8, and the C-terminal polyclonal anti-tau antibody K9JA, and used for confocal microscopy. The scale bar is 50 µm. (**B**) Graphs show the average intensity of AT8-detected p-Tau, K9JA-detected total Tau, and the ratio of p-Tau/total Tau. Data obtained for each condition are normalized to the medium control. The values shown in graphs are the average of triplicate wells for each treatment ± SEM. Results shown in (**A**)-(**B**) are representative of at least two independent experiments.

Jin, O'Nuallain *et al.*, Supplementary Figure 12

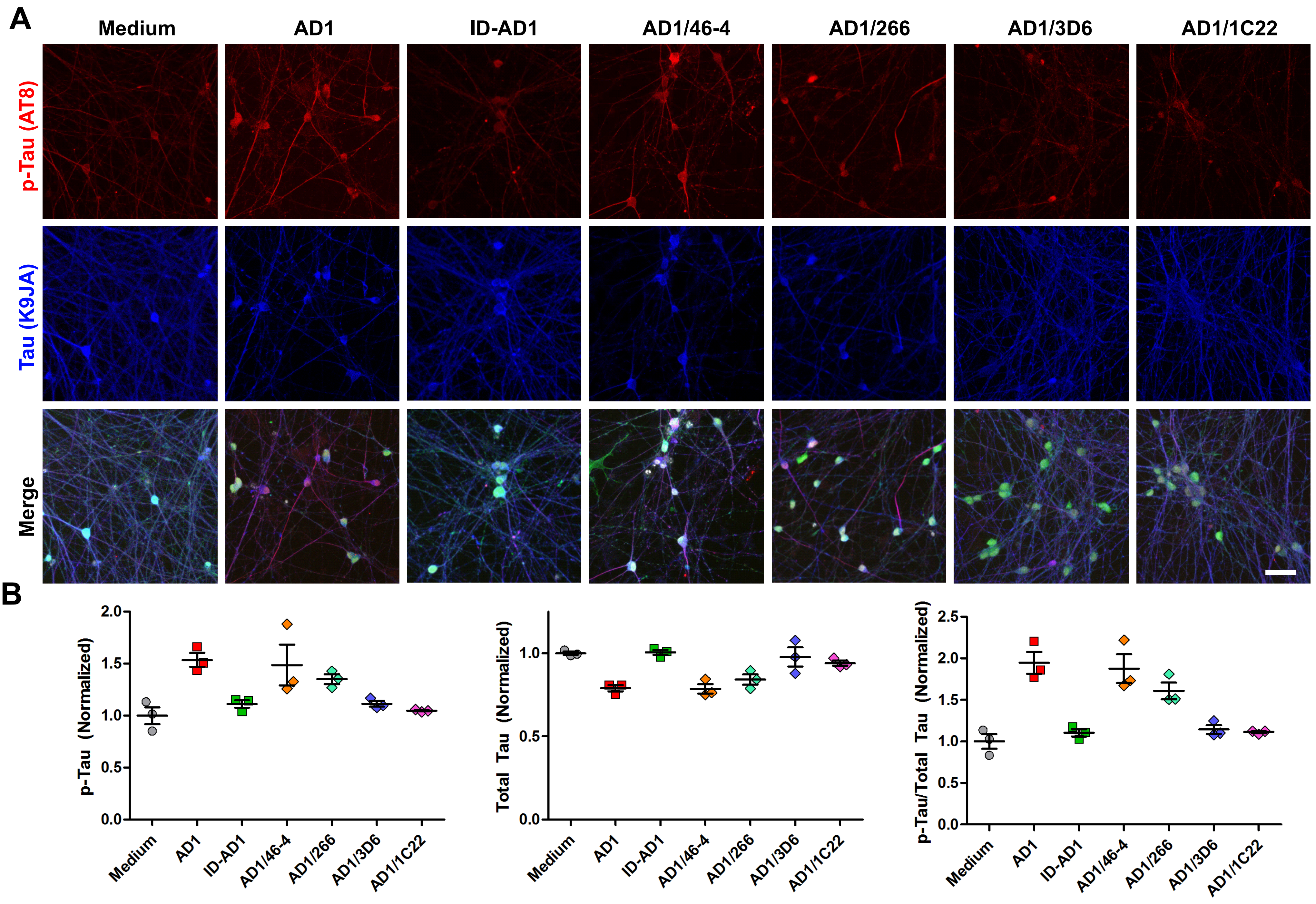

Supplement: Supplementary file 1 — Supplementary Information [file 41467_2018_5068_MOESM1_ESM.pdf]
